# Meta-transcriptomic analysis reveals the geographical expansion of known sugarbeet-infecting viruses and the occurrence of a novel virus in sugarbeet in the United States

**DOI:** 10.3389/fpls.2024.1429402

**Published:** 2024-08-30

**Authors:** Chinnaraja Chinnadurai, Nathan A. Wyatt, John J. Weiland, Oliver T. Neher, Joe Hastings, Mark W. Bloomquist, Chenggen Chu, Ashok K. Chanda, Mohamed Khan, Melvin D. Bolton, Vanitharani Ramachandran

**Affiliations:** ^1^ Department of Plant Pathology, North Dakota State University, Fargo, ND, United States; ^2^ United States Department of Agriculture, Agricultural Research Service, Edward T. Schafer Agricultural Research Center, Fargo, ND, United States; ^3^ Sugarbeet Research, Amalgamated Sugar Company, Boise, ID, United States; ^4^ Agriculture Department, American Crystal Sugar Company, Moorhead, MN, United States; ^5^ Agriculture Department, Southern Minnesota Beet Sugar Cooperative, Renville, MN, United States; ^6^ Department of Plant Pathology, University of Minnesota, St. Paul, MN, United States; ^7^ Northwest Research and Outreach Center, University of Minnesota, Crookston, MN, United States

**Keywords:** RNA sequencing, virome, rhizomania, meta-transcriptomic, sugarbeet, *Beta vulgaris*

## Abstract

In this study, meta-transcriptome sequencing was conducted on a total of 18 sugarbeet (*Beta vulgaris* L. subsp*. vulgaris*) sample libraries to profile the virome of field-grown sugarbeet to identify the occurrence and distribution of known and potentially new viruses from five different states in the United States. Sugarbeet roots with symptoms resembling rhizomania caused by beet necrotic yellow vein virus (BNYVV), or leaves exhibiting leaf-curling, yellowing to browning, or green mosaic were collected from the sugarbeet growing areas of California, Colorado, Idaho, Minnesota, and North Dakota. *In silico* analysis of *de novo* assembled contigs revealed the presence of nearly full-length genomes of BNYVV, beet soil-borne virus (BSBV), and beet soil-borne mosaic virus (BSBMV), which represent known sugarbeet-infecting viruses. Among those, BNYVV was widespread across the locations, whereas BSBV was prevalent in Minnesota and Idaho, and BSBMV was only detected in Minnesota. In addition, two recently reported *Beta vulgaris* satellite virus isoforms (BvSatV-1A and BvSatV-1B) were detected in new locations, indicating the geographical expansion of this known virus. Besides these known sugarbeet-infecting viruses, the bioinformatic analysis identified the widespread occurrence of a new uncharacterized *Erysiphe necator-*associated abispo virus (En_abispoV), a fungus-related virus that was identified in all 14 libraries. En_abispoV contains two RNA components, and nearly complete sequences of both RNA1 and RNA2 were obtained from RNASeq and were further confirmed by primer-walking RT-PCR and Sanger sequencing. Phylogenetic comparison of En_abispoV isolates obtained in this study showed varying levels of genetic diversity within RNA1 and RNA2 compared to previously reported isolates. The undertaken meta-transcriptomic approach revealed the widespread nature of coexisting viruses associated with field-grown sugarbeet exhibiting virus disease-like symptoms in the United States.

## 1 Introduction

Sugarbeet (*Beta vulgaris* L. subsp*. vulgaris*), a sucrose-accumulating root crop that contributes one-fourth of the world’s sugar, is cultivated in Europe, Asia, Mediterranean countries, and parts of North America (Biancardi et al., 2010). In the United States (U. S.), sugarbeet is grown in four regions covering 11 states viz., the Great Lakes region (Michigan) to the east of the Mississippi River, the upper Midwest (Minnesota and North Dakota), the Great Plains (Colorado, Montana, Nebraska, and Wyoming), and the Far West (California, Idaho, Oregon, and Washington), where approximately 1.5 million acres are grown annually (https://www.ers.usda.gov). Sugarbeet production is affected by a number of viral diseases. Among the viral diseases of sugarbeet, rhizomania, caused by the *Beet necrotic yellow vein virus* (BNYVV, genus *Benyvirus*), is a leading global virus disease of sugarbeet (Tamada, 1999; Tamada and Baba, 1973; Rush et al., 2006). In the U. S., rhizomania was first reported in California in 1983, and shortly after, had spread to all sugarbeet-growing regions of the U. S (Duffus et al., 1984; Weiland et al., 2019; Wisler et al., 1997). BNYVV is transmitted by *Polymyxa betae*, a plasmodiophoromycete vector that can survive long periods in the soil under favorable conditions (Fujisawa and Sugimoto, 1977). The same *P. betae* vector also transmits three other soil-borne viruses *Beet soil-borne mosaic virus* (genus *Benyvirus*), Beet soil-borne virus (genus *Pomovirus*), and *Beet virus Q* (genus *Pomovirus*) in sugarbeet (Meunier et al., 2003). Curly top is another yield-limiting viral disease of sugarbeet. The disease is caused by ssDNA *Beet curly top virus* (genus *Curtovirus*). Beet curly top virus (BCTV) is transmitted by the beet leafhopper, *Circulifer tenellus*, and there are 11 strains of BCTV reported that cause curly top disease in sugarbeet (Strausbaugh et al., 2017; Teng et al., 2010). Virus yellows is caused by a complex of viruses including *Beet mild yellowing virus* (genus *Polerovirus*), *Beet chlorosis virus* (genus *Polerovirus*), and *Beet yellows virus* (genus *Closterovirus*). Beet mild yellowing virus (BMYV) and Beet chlorosis virus (BChV) are typically transmitted by the green peach aphid *Myzus persicae* (100%), and also by *Macrosiphum euphorbiae* (83 - 98%) (Hossain et al., 2021; Kozłowska-Makulska et al., 2009). BYV has been reported to be transmitted by more than 20 aphid species, with *Myzus persicae* and *Aphis fabae* emerging as the primary natural carriers (German-Retana et al., 1999). *Beet mosaic virus* (BtMV) is an aphid-transmissible potyvirus causing mosaic symptoms on leaves and can exacerbate virus yellows disease when co-infecting sugarbeet with that complex (Wintermantel, 2005).

The advent of next-generation high-throughput sequencing (HTS) technologies has offered the opportunity to discover an unprecedented number of viruses that have advanced our knowledge of virus diversity in many agricultural crop plants. Prior to the HTS era, etiological investigations of economically important virus-like diseases led to the discovery of new viruses in crops. One approach to HTS-enabled virus discovery exploits RNA-silencing, which is an anti-viral defense mechanism that operates in plants to combat viruses. Viruses can activate the plant RNA silencing defense system and become targets of virus-derived small RNAs (vsiRNAs), a hallmark of the RNA silencing phenomenon (Kwon et al., 2020; Vanitharani et al., 2005; Saumet and Lecellier, 2006). In the past, HTS of vsiRNAs enabled the identification of new viruses; however, virus assembly was tedious due to the short-read size of small RNA. With the advancement of HTS technology, RNA-Seq with an increased read length is being widely used to capture the ‘virome’ in a wide range of environmental and biological samples (Kamitani et al., 2019; Lai et al., 2022). In this way, researchers can understand at greater depth the virus spectrum in plants, which in turn facilitates new ways to study mixed infections and understand the possible synergistic or antagonistic interactions that might lead to disease.

The study of viromes has gained increasing attention in virus research, and it has been documented in various crops and fruits (Jo et al., 2017, 2018, 2020a, b; Pecman et al., 2017). RNA-Seq of rhizomania-infected sugarbeet roots led to the isolation of a U. S. strain of BNYVV, a novel *B. vulgaris* alphanecrovirus (BvANV-1) and a putative *B. vulgaris* satellite virus (BvSatV) in addition to the known soil-borne viruses BSBMV, BSBV, beet black scorch virus (BBSV), and BVQ (Weiland et al., 2020). Recently, using meta-transcriptomic sequencing of sugarbeet with stunted growth, Ramachandran and co-workers identified the presence of *Tomato bushy stunt virus* that belongs to the genus *Tombusvirus*, and BNYVV (Ramachandran et al., 2023a). Further, a study on table beet with hairy roots and shorter petiole symptoms revealed the occurrence of the *Pepper yellow dwarf* strain of BCTV and *Spinach curly top Arizona virus* (Ramachandran et al., 2023b). Despite these investigations, the thorough examination of viromes related to sugarbeet roots and leaves is limited.

In this study, we explored the natural diversity of viruses in 18 field-grown sugarbeet samples collected from five U.S. states that were symptomatic for rhizomania, root diseases, or other virus-like foliar diseases that resembled curly top and virus yellows by subjecting them to HTS. The analysis of HTS data revealed the occurrence and distribution of known sugarbeet-infecting soil-borne and foliar viruses such as BNYVV, BSBMV, BSBV, and BCTV. Interestingly, the newly reported BvSatV from Minnesota (Weiland et al., 2020) was found nearly nationwide and was identified in Colorado, Idaho, and North Dakota, indicating a larger distribution within the U.S. The number of co-infections and differences in virus titers revealed through RNA-Seq was further confirmed using RT-PCR analysis and Sanger sequencing, adding a measurable component to our analysis. Our HTS analysis resulted in the identification of a virus related to a fungus-infecting virus, *Erysiphe necator-*associated abispo virus (En_abispoV), for the first time in sugarbeet. Furthermore, this virus was detected in multiple sugarbeet growing locations within the U. S. The nearly full-length genome sequences of RNA1 and RNA2 of the En_abispoV were determined using primer walking, RT-PCR, and sequence confirmation by Sanger sequencing which validated the HTS results. The identification of this mycovirus-related virus in sugarbeet in multiple locations suggests an association of a fungal host with sugarbeet across the nation.

## 2 Materials and methods

### 2.1 Sample collection

Field-grown sugarbeet roots samples with rhizomania symptoms and leaf samples with varying virus disease-like symptoms of leaf-curling, yellowing to browning, or green mosaic were collected from the production fields of five different states in the U. S. (California, Colorado, Idaho, Minnesota, and North Dakota). Sample details as described in **Table 1**. Each beet sample represents a pool of hairy roots obtained from four to eight beets with rhizomania symptoms. Each leaf sample represents portions of leaves obtained from four to eight sugarbeet plants exhibiting leaf symptoms resembling virus diseases. The samples from Minnesota and North Dakota were obtained in coordination with the agriculturalists of the American Crystal Sugar Company (Moorhead, MN), Minn-Dak Farmers’ Cooperative (Wahpeton, ND), and Southern Minnesota Beet Sugar Cooperative (Renville, MN). The agriculturalists of Amalgamated Sugar Company (Boise, Idaho) and Spreckels Sugar Company (Brawley, CA) provided the samples from Idaho and the Imperial Valley of California, respectively, and leaf samples were collected from Colorado.

**Table 1:** Sample details are given with corresponding RNA sequencing read counts, reads mapped with sugarbeet genome and the number of known and new viruses identified in each sample.

Sample ID	Plant material	Location	Symptoms	Raw reads	Cleaned reads	RefBeet mapped reads	Major viral hits (known)	New virus (This study)
LS-1	Sugarbeet leaf	Minnesota	Leaf curling	90845002	82438928	73554506	No hit	En_abispoV
LS-2	Sugarbeet leaf	Minnesota	Leaf curling	78246166	68054502	63837831	BNYVV, BSBMV, BSBV, BvSatV	En_abispoV
LS-3	Sugarbeet leaf	Minnesota	Leaf browning	52053897	50416306	47541014	No hit	En_abispoV
LS-4	Sugar beet leaf	Minnesota	Pale yellow leaf	74067691	71694196	70513376	BvSatV	En_abispoV
LS-5	Sugar beet leaf	Colorado	Virus yellows like	55453681	53058786	49119145	BNYVV, BSBV, BvSatV	None
LS-6	Sugar beet leaf	Colorado	Virus yellows like	61434972	57460862	50003927	BNYVV, BSBV, BvSatV	En_abispoV
LS-7	Sugar beet leaf	Colorado	Virus yellows like	57982821	54835076	52751266	BNYVV	En_abispoV
LS-8	Sugar beet leaf	Colorado	Virus yellows like	92781163	89363196	78220865	BNYVV	En_abispoV
LS-9	Sugar beet leaf	Minnesota	Green mosaic	61067543	57557070	57079361	BvSatV	None
BS-1	Sugar beet root	California	Rhizomania-like	77286055	76421130	66304588	BNYVV	None
BS-2	Sugar beet root	Minnesota	Rhizomania-like	95343104	91104798	87562039	BNYVV, BSBMV, BSBV, BvSatV	En_abispoV
BS-3	Sugar beet root	Minnesota	Rhizomania-like	96269692	87728010	83004296	BNYVV, BSBMV, BvSatV, BvANV-1	En_abispoV
BS-4	Sugar beet root	Minnesota	Rhizomania-like	131704187	113079702	108888832	BNYVV, BSBMV, BSBV, BvSatV	En_abispoV
BS-5	Sugar beet root	Minnesota	Rhizomania-like	111323704	98611082	74156692	BNYVV, BSBMV, BSBV, BvSatV	En_abispoV
BS-6	Sugar beet root	Minnesota	Rhizomania-like	71429229	60972252	53756433	BNYVV, BSBMV, BSBV, BvSatV	En_abispoV
BS-7	Sugar beet root	North Dakota	Rhizomania-like	99144096	84626106	72707202	BNYVV, BSBMV, BSBV, BvSatV	En_abispoV
BS-8	Sugar beet root	Minnesota	Rhizomania-like	77043522	67745390	64567582	BNYVV, BvSatV	En_abispoV
BS-9	Sugar beet root	Idaho	Rhizomania-like	74691654	65889374	59504029	BNYVV, BSBV, BvSatV, BCTV	None

### 2.2 High-throughput sequencing and data analysis

**Figure 1:**
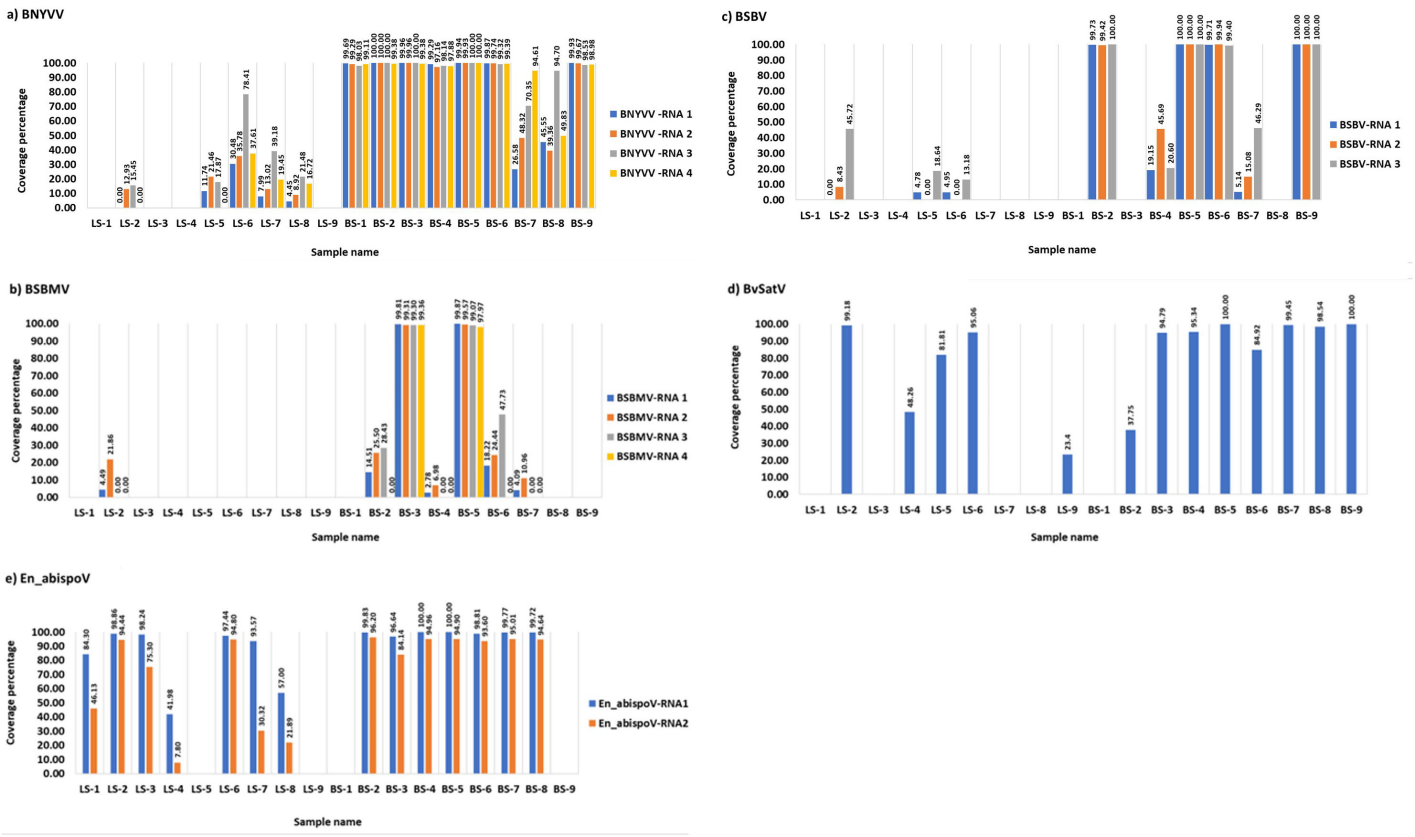
The coverage percentage in the bar diagram represents the total length of RNA segments obtained from bowtie2 mapping for major known and newly identified viruses. Seven root libraries for BNYVV **(A)**, two for BSBMV **(B)**, four for BSBV **(C)**, eight for BvSatV **(D)**, and eight for En_abispoV **(E)** were found with more than 90% genome coverage in their RNA segments. Reads mapped to BCTV in beet sample BS-9 (Idaho) covered 37.04% of the genome and beet sample BS-3 (Minnesota) was found to have 62.02% coverage for BvANV-1.

Total RNA was extracted from the hairy-root tissue of beet samples or from the symptomatic leaf samples obtained from the harvested sugarbeet plants using the RNeasy plant mini kit (Qiagen, USA) according to the manufacturer’s instructions. For RNA-Seq, 18 libraries were constructed using the Illumina Ribo-Zero Plus rRNA Depletion Kit (Illumina, CA) and sequenced on the Illumina NovaSeq 6000 platform at Novogene Corporation Inc (Sacramento, California). Paired-end reads of each sample were collected from Novogene Corporation Inc. Adapter sequences were trimmed, and low-quality reads (Q<30) were filtered using Trimmamotic v0.39 (Bolger et al., 2014). Quality filtering eliminated 1.12% to 14.64% of raw reads, and approximately 75.2% to 98.35% of clean reads (**Table 1**) were mapped against RefBeet-1.2.2 (GCA_00511025.2), a reference sugarbeet genome retrieved from GenBank, for host genome mapping using Bowtie2 v2.5.1. Samtools v1.17 was used to produce mapping statistics. Bamtools v2.5.2 was used to convert BAM files into FASTQ files. The remaining unmapped reads from RefBeet-mapping were *de novo* assembled to generate contigs using SPAdes genome assembler v3.15.5 (Bankevich et al., 2012). Assembled contigs were subjected to BLASTn search with an E-value cutoff of set to 1e-10 using blast+ v2.13.0. Geneious Prime 2023.1.1 was used to construct complete genome fragments of beet viruses using contigs larger than 150 nts in size generated from *de novo* assembly. The genome coverage percentage was obtained after mapping the reads to each of the RNA components of individual viruses (**Figure 1**) using the Burrows-Wheeler transform algorithm (Li and Durbin, 2009). The raw sequencing data for each sample were deposited in the National Centre for Biotechnology Information (NCBI)-Sequence Read Archive (SRA) database with accession numbers under the BioProject number PRJNA1107161.

### 2.3 RT-PCR analysis and validation of HTS results

To validate the obtained HTS results, RT-PCR was performed using a one-step RT-PCR kit (Qiagen, MA, USA) for individual samples to confirm the presence of sugarbeet infecting viruses using primers to amplify target regions on the various genomic RNA components for each virus. The primer details used for the amplification of each virus are given in **Supplementary Table 1** with primer numbers as follows: BNYVV, 1 to 8; BSBV, 9-14; BSBMV, 15-22; BvSatV, 23 to 24; BvANV-1, 25 to 26; and En_abispoV, RNA1 (27 to 28) and RNA2 (39 to 40). The RT-PCR mix contained 4 µl of 5x one-step RT-PCR buffer, 1 µl of 10 mM dNTP mix, 1 µl of 10 mM each forward and reverse primers, 20 U of RNase inhibitor (Invitrogen, USA), 1 µl of one-step RT-PCR enzyme mix, and 100 ng of total RNA and adjusted with nuclease-free water to a final volume of 20 µl. The PCR conditions differed in annealing temperature with a range between 52-58°C for each virus fragment based on the Tm values of the primer pairs. PCR conditions were as follows: 50°C for 60 mins, 95°C for 15 mins, followed by 35 cycles of 95°C for 40 sec, 52-58°C for 40 sec, and 72°C for 45 sec, and then final primer extension at 72°C for 15 mins. All PCR amplicons were gel purified using a QIAquick Gel Extraction kit (Qiagen, MA, USA) and Sanger sequenced from both ends (Eurofins Genomics, KY, USA). A BLASTn search on NCBI was used to confirm the presence of viruses using sequencing results.

### 2.4 Genome characterization of *Erysiphe necator-*associated abispo virus

In addition to the contigs extracted from *de novo* assembly, primer walking strategies were followed using RT-PCR to amplify the complete genome of En_abispoV. Four samples BS-2, LS-1, LS-2 (Minnesota), and LS-6 (Colorado) were selected to obtain the complete genome sequences. Six primer pairs targeting contiguous overlapping genome fragments of RNA1 (Primer numbers 27 to 38; **Supplementary Table 1**) and five primer pairs for RNA2 (Primer numbers 39 to 48; **Supplementary Table 1**) were designed using a Multiple Primer Analyzer (ThermoFisher Scientific, USA) based on closely matched reference genome sequences (MN611695.1 and MN611694.1) retrieved from BLASTn analysis. A one-step RT-PCR assay (Qiagen, MA, USA) was performed in Pro-Flex PCR systems (Applied Biosystems, USA). The PCR mix contained 4 µl of 5x one-step RT-PCR buffer, 1 µl of 10 mM dNTP mix, 1 µl of 10 mM forward and reverse primers, 20 U of RNase inhibitor (Invitrogen, USA), 1 µl of one-step RT-PCR enzyme mix, and nuclease-free water to a final volume of 20 µl. The PCR conditions were as follows; 50°C for 60 mins, 95°C for 15 mins, followed by 35 cycles of 95°C for 40 sec, 52-56°C for 40 sec, and 72°C for 45 sec, and then final primer extension at 72°C for 10 mins. All PCR amplicons were gel purified using a QIAquick Gel Extraction kit (Qiagen, MA, USA) and the sequence was confirmed using Sanger sequencing from both directions (Eurofins Genomics, KY, USA). Geneious Prime 2023.1.1 was used to assemble the full genome sequences of RNA1 and RNA2 obtained through the Sanger sequencing.

ORF finder from the NCBI was used to find the candidate open reading frames in the two RNA sequences. The Conserved Domain Database (CDD)-NCBI, Pfam 35.0, and InterPro 91.0 at the European Molecular Biology Laboratory- European Bioinformatics Institute (EMBL-EBI) were used to confirm the conserved domain of protein sequences. The genome annotation transfer utility (GATU) (Tcherepanov et al., 2006) was used to annotate the RNA1 and RNA2 of newly identified virus isolates in this study with *Erysiphe necator-*associated abispo virus 8 isolate PMS7_214 segment RNA1, complete sequence (MN611695.1) and *Erysiphe necator-*associated abispo virus 7 isolate PMS5_242 segment RNA2, complete sequence (MN611694.1) as reference sequences, respectively.

### 2.5 Phylogenetic analysis

Phylogenetic analysis was performed for BvSatV and En_abispoV. For BvSatV, RNA sequences from five beet samples from Minnesota (BS-3, BS-4, BS-5, BS-6, and BS-8), one beet sample each from North Dakota (BS-7) and Idaho (BS-9), and two leaf samples from Colorado (LS-5 and LS-6), and one leaf sample from Minnesota (LS-2) were used. For the newly identified En_abispoV, RNA1 and RNA2 sequences from six beet samples from Minnesota (BS-2, BS-3, BS-4, BS-5, BS-6, and BS-8), one beet sample from North Dakota (BS-7), three leaf samples from Minnesota (LS-1, LS-2, and LS-3), and two leaf samples from Colorado (LS-6 and LS-7) were used in phylogenetic analysis. For En_abispoV, virus isolates that matched En_abispoV, including Grapevine-associated RNA virus 4 (GVRV), *Sclerotinia sclerotiorum* virga-like virus 1, and *Agaricus bisporus* virus 16 were used in the analysis. In total, 14 isolates related to En_abispoV covering the entire ORF region from different geographical regions, and all three available BvSatV isolates reported from the U.S. were retrieved from the NCBI GenBank and used in the phylogenetic analysis. Sequence alignment was performed using the ClustalW multiple alignment tool and the sequence identity matrix was obtained using BioEdit Sequence Alignment Editor 7.2.5 software (Hall et al., 2011). All the aligned sequences were trimmed to equal length and used for the phylogenetic tree construction. Maximum-likelihood phylograms were constructed following LG with the Freq.(+F) substitution model and the Nearest-Neighbor-Interchange (NNI) ML Heuristic method with 1000 bootstrap replications using MEGA 11.0.13 software (Tamura et al., 2021).

## 3 Results

### 3.1 Identification and geographical expansion of known sugarbeet-infecting viruses

**Figure 2:**
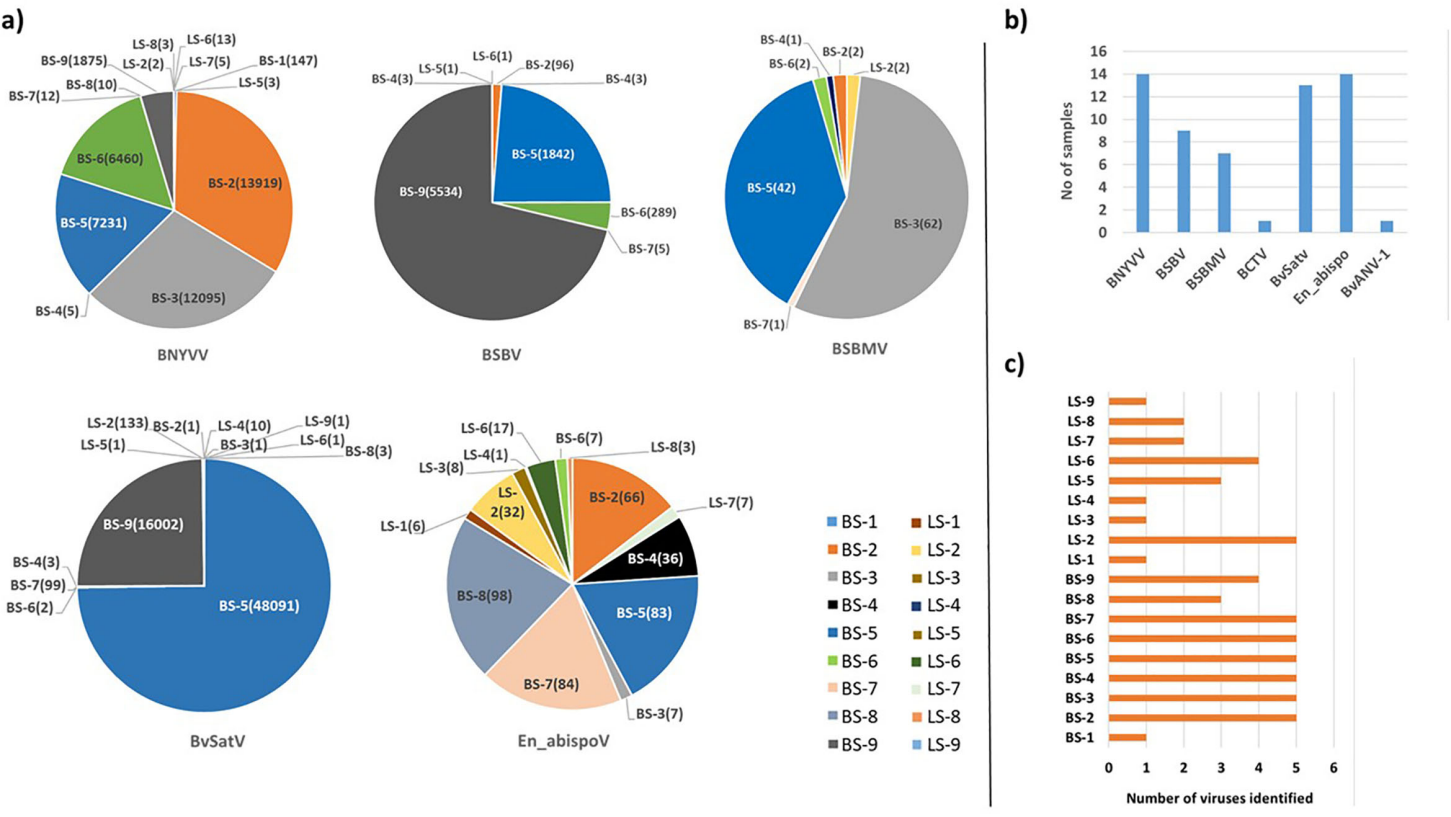
**(A)** The total number of contigs obtained in RNA sequencing for major known and newly detected viruses in root and leaf samples of sugar beet from five different states in the U.S. Contig numbers are given in parentheses (). **(B)** Number of samples found with each virus. **(C)** Number of viruses identified in each sample. In 14 libraries, BNYVV and En_apispoV emerged as prevalent viruses, while in 13 libraries, BvSatV was identified as the second most common. BCTV was identified only in beet sample BS-9 from Idaho with 3 contigs and BvANV-1 was identified in beet sample BS-3 from Minnesota with 4 contigs.

Meta-transcriptomic analysis of total RNA isolated from sugarbeet leaf samples exhibiting virus disease-like symptoms of leaf-curling, yellowing to browning, or green mosaic, and root samples with rhizomania symptoms yielded 52 to 131 million raw reads across the 18 libraries. Among the libraries, the known and historically most commonly occurring sugarbeet-infecting viruses, BNYVV, BSBV, and BSBMV were identified as the major viral hits (**Table 1**). Overall, the virus-specific contigs were found more in the beet root samples than in the leaf samples. The number of virus-associated contigs ranked higher for BNYVV compared to BSBV and BSBMV (**Figure 2**). BNYVV-specific contigs were found in 14 out of 18 libraries, and among these nine libraries constructed from root samples had contigs specific to BNYVV RNA1, RNA2, RNA3, and RNA4 (**Supplementary Table 2**). Although BNYVV was detected in five leaf-derived samples, overall, the number of contigs was low and did not represent all four RNA components of BNYVV except in one sample, LS-6. None of the 14 libraries revealed contigs matching to RNA5 of BNYVV which is consistent with the previously observed absence of RNA5 in the U.S. The highest number of BNYVV-specific contigs was found in BS-2, 13919 from Minnesota, while BS-9 from Idaho had 1875 contigs, and BS-1 from California recorded 147 contigs (**Figure 2**). The genome coverage of BNYVV on RNA1 through RNA4 was observed to be more than 97% in seven root libraries originating from Minnesota, Idaho, and California (**Figure 1**). Details of BLAST results of the assembled consensus sequences obtained for major viruses from each library are provided in **Supplementary Table 3**.

The contigs specific to BSBV were found in nine (six beet root and three leaf) samples. The maximum number of contigs corresponding to BSBV was found in BS-9 (5534) from Idaho, followed by BS-5 (1842) and BS-6 (289) from Minnesota (**Figure 2**; **Supplementary Table 2**). Mapping of BSBV-specific reads representing all three genomic RNAs in all three libraries resulted in more than 99% genome coverage of the viral segments (**Figure 1**). In the case of BSBMV, which is only reported in the U. S., contigs representing all four BSBMV RNA segments were identified in two root libraries, BS-3 and BS-5, from Minnesota with genome coverage ranging from 97.97% to 99.87% (**Figure 1**). A reduced number of contigs representing partial sequences of BSBMV was found in the BS-2, BS-4, BS-6, and BS-7 libraries, and in the leaf-derived one (**Supplementary Table 2**). Among the major sugarbeet-infecting foliar viruses, BCTV, which is commonly found in the western region of the U.S. was detected in BS-9 from Idaho; however, the coverage was only 37%, representing the partial genome of BCTV.

The recently reported BvSatV from Minnesota (Weiland et al., 2020) was detected in 13 out of 18 libraries. BS-5 from Minnesota was found to possess a large number of contigs, 48091 for this virus, and the second largest was in the library BS-9 from Idaho with 16002 contigs (**Figure 2**). BvSatV was detected in the leaf sample libraries, LS-5 and LS-6, from Colorado, and LS-2, LS-4, and LS-9 from Minnesota. The BvSatV isoform BvSatV-1B was found only in two libraries, BS-9 from Idaho and LS-2 from Minnesota, and the remaining 11 libraries mainly possessed the isoform BvSatV-1A. Overall, more than 80% genome coverage for BvSatV was found in seven root- and three leaf-libraries (**Figure 1**). The identification of BvSatV for the first time in Idaho and Colorado shows the presence of the virus in sugarbeet growing areas other than Minnesota. Another recently discovered BvANV-1 (Weiland et al., 2020) was detected only in BS-3 from Minnesota with 62% genome coverage. In addition to these significant known sugarbeet-infecting viruses, contigs representing *Beta vulgaris* mitovirus and beet cryptic virus were observed in many beet root and leaf samples. Additionally, a previously reported *Lettuce chlorosis virus* (Wisler et al., 1997) was detected in library BS-1, a sample originating from California.

Altogether, BNYVV was detected in samples originating from all the locations of California, Colorado, Idaho, Minnesota, and North Dakota, and BSBV was detected in four locations except California, whereas BSBMV presence was found only in the Minnesota and North Dakota areas. Interestingly, BvSatV isoforms (BvSatV-1A and BvSatV-1B), which were recently found in Minnesota, were detected in the samples obtained from Colorado, Idaho, and North Dakota, indicating the expanded occurrence of BvSatV in new sugarbeet growing areas in addition to Minnesota. Comparative analysis revealed less sequence diversity for BNYVV, BSBV, BSBMV, and BvSatV. The corresponding RNA components shared greater than 95% sequence identity with the reference genomes and hence these previously characterized sugarbeet-infecting viruses were not subjected to detailed analysis in this study.

### 3.2 Identification of a putative mycovirus-related virus naturally occurring in sugarbeet

Other than the known sugarbeet-infecting viruses, a relatively large number of contigs related to the fungus-associated viruses such as *Erysiphe necator*-associated abispo virus, *Erysiphe necator-*associated narnavirus*, Erysiphe necator*-associated ourmia-like virus, and *Plasmopara viticola* lesion-associated ourmia-like virus were detected in both beet root and leaf sample libraries. Among these mycoviruses, contigs related to *Erysiphe necator*-associated abispo virus (designated En_abispoV) were taken into detailed genome characterization and phylogenetic analysis since En_abispoV is an understudied virus and no publications are available on this virus other than GenBank sequence information. The contigs that were closely related to En_abispoV were detected in 14 out of 18 libraries including both leaf and root samples (**Figure 2**; **Supplementary Table 2**). BLASTn search analysis identified contigs matching to *Erysiphe necator*-associated abispo virus 8 isolate PMS7_214 segment RNA1, complete sequence (MN611695.1), with 95.4% to 97.18% nucleotide identity, and *Erysiphe necator*-associated abispo virus 7 isolate PMS5_242 segment RNA2, complete sequence (MN611694.1), with 93.2% to 96.87% nucleotide identity. The genome coverage of both En_abispoV RNA1 and RNA2 was greater than 90% in all root samples that were positive for En_abispoV except BS-3, which showed 84% coverage for RNA2, and all these root samples originated from Minnesota and North Dakota (**Figure 1**). The contigs corresponding to En_abispoV were undetectable in samples BS-1 and BS-9 which represented California and Idaho, respectively. The contigs matching En_abispoV RNA1 and RNA2 were identified in seven out of nine leaf samples that originated from the Minnesota and Colorado regions; however, the overall degree of genome coverage was variable (**Figure 1**). Unlike the major known sugarbeet-infecting viruses, the En_abispoV RNA1 and RNA2 specific contigs were detected both in the leaf and root samples of sugarbeet plants. Further, this study revealed the existence of En_abispoV RNA1 and RNA2 in Minnesota, North Dakota, and Colorado. It is worth noting that contigs representing En_abispoV RNA1 and RNA2 were not detected in the samples obtained from California and Idaho (**Table 1**).

Among the identified viruses, the well-known BNYVV, and the newly identified, En_abispoV were widespread as they were represented in 14 of the 18 libraries. BvSatV represents the second most prevalent viral pathogen detected (13 libraries) followed by BSBV (detected in nine libraries), and BSBMV being found in seven libraries (**Figure 2**). Overall, these results indicate the existence of multiple viruses in field samples and were more prevalent in the roots compared to the leaves of sugarbeet plants (**Figure 2**).

### 3.3 Validation of HTS-identified viruses using RT-PCR

**Figure 3:**
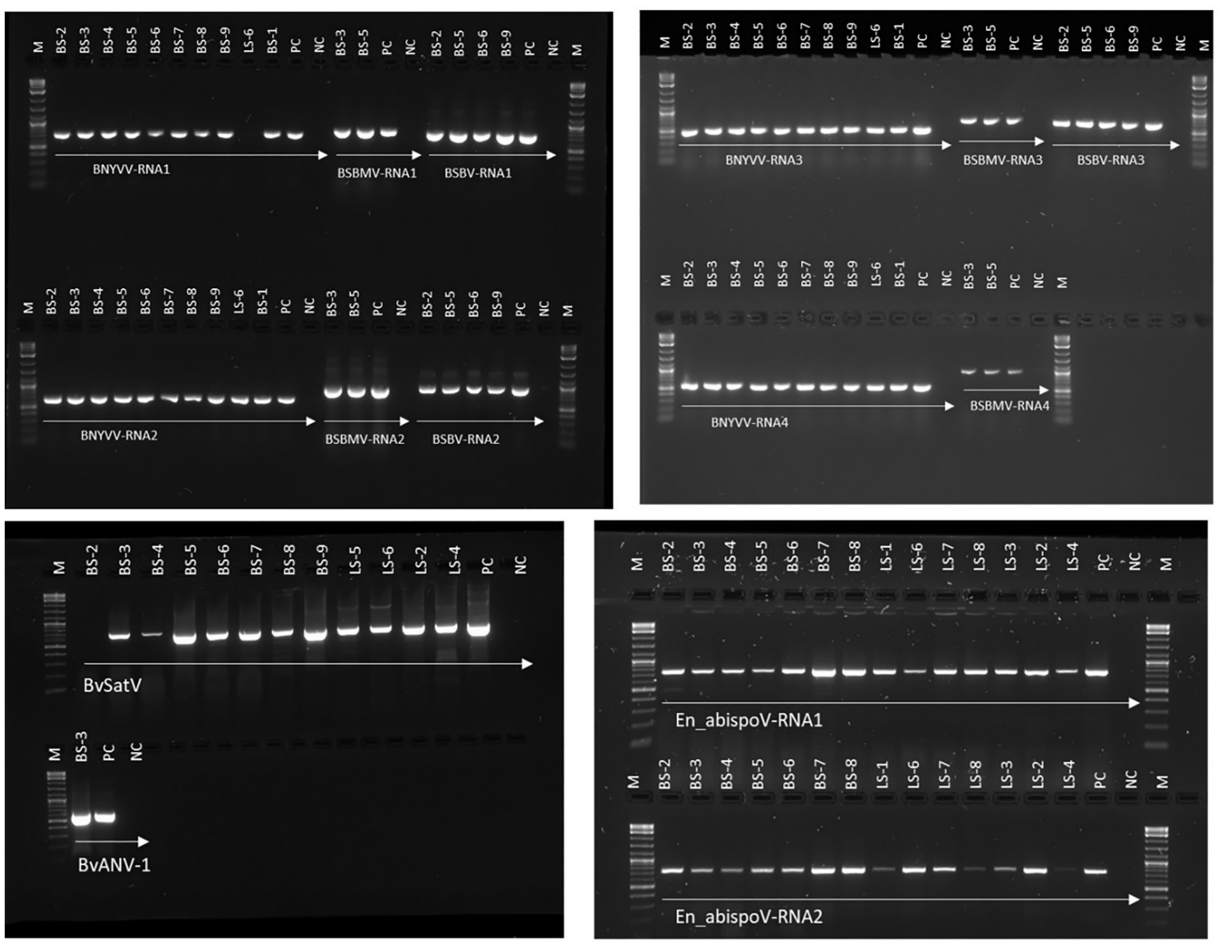
RT-PCR-based validation for major sugar beet infecting viruses identified in RNA sequencing from sugar beet samples. Target genome sizes: BNYVV-RNA1, 729 bp; BNYVV-RNA2, 628 bp; BNYVV-RNA3, 504 bp; BNYVV-RNA4, 567 bp; BSBMV-RNA1, 828 bp; BSBMV-RNA2, 912 bp; BSBMV-RNA3, 738 bp; BSBMV-RNA4, 962 bp; BSBV-RNA1,772 bp; BSBV-RNA2,922 bp; BSBV-RNA3,704 bp; BvSatV, 706 bp; BvANV-1,644 bp; En_abispoV-RNA1,773 bp; and En_abispoV-RNA2, 766 bp. M, 1kb plus DNA ladder; PC, positive control; NC, negative control. BNYVV-RNA1 in LS-6 and BvSatV in BS-2 failed to amplify in the RT-PCR reaction and a low coverage might be the reason behind the failure of amplification.

To validate the meta-transcriptomic results, RT-PCR analysis was carried out to confirm the presence of three common infecting viruses, BNYVV, BSBMV, and BSBV; the two recently discovered BvSatV isoforms and BvANV-1; and the newly identified En_abispoV (**Figure 3**). Using RT-PCR, the target genome fragments were amplified for BNYVV RNA1 to RNA4 in all nine root samples, BS-1 through BS-9, as described in **Table 1**. Although contigs representing BNYVV were detectable in leaf samples, amplification of the genome using RT-PCR was difficult due to low coverage. For example, in sample LS-6, RT-PCR amplified RNA2, 3, and 4, but was unable to amplify RNA1.

For BSBMV, target genome fragments were amplified for RNA1 to RNA4 in two root samples from Minnesota, and for the BSBV target regions were amplified for RNA1-RNA3 in four root samples, of which three samples were from Minnesota and one from Idaho (**Figure 3**). BvSatV was amplified in seven root (BS-3 to BS-9) and four leaf samples (LS-2, LS-4 to LS-6) (**Table 1**). RT-PCR failed to amplify BvSatV in BS-2 due to low coverage. BvANV-1 was detected only in BS-3 from Minnesota, and in that sample, the target fragment of BvANV-1 was amplified (**Figure 3**). Due to low coverage, BCTV was not subjected to PCR-based confirmation in sample BS-9. En_abispoV RNA1 and RNA2 were successfully amplified from seven root samples, BS-2 to BS-8, and in seven leaf samples, LS-1 to 4 and LS-6 to 8, except for LS-1 and LS-4 from Minnesota and LS-8 from Colorado where mild amplification was encountered for RNA2 (**Figure 3**).

**Figure 4:**
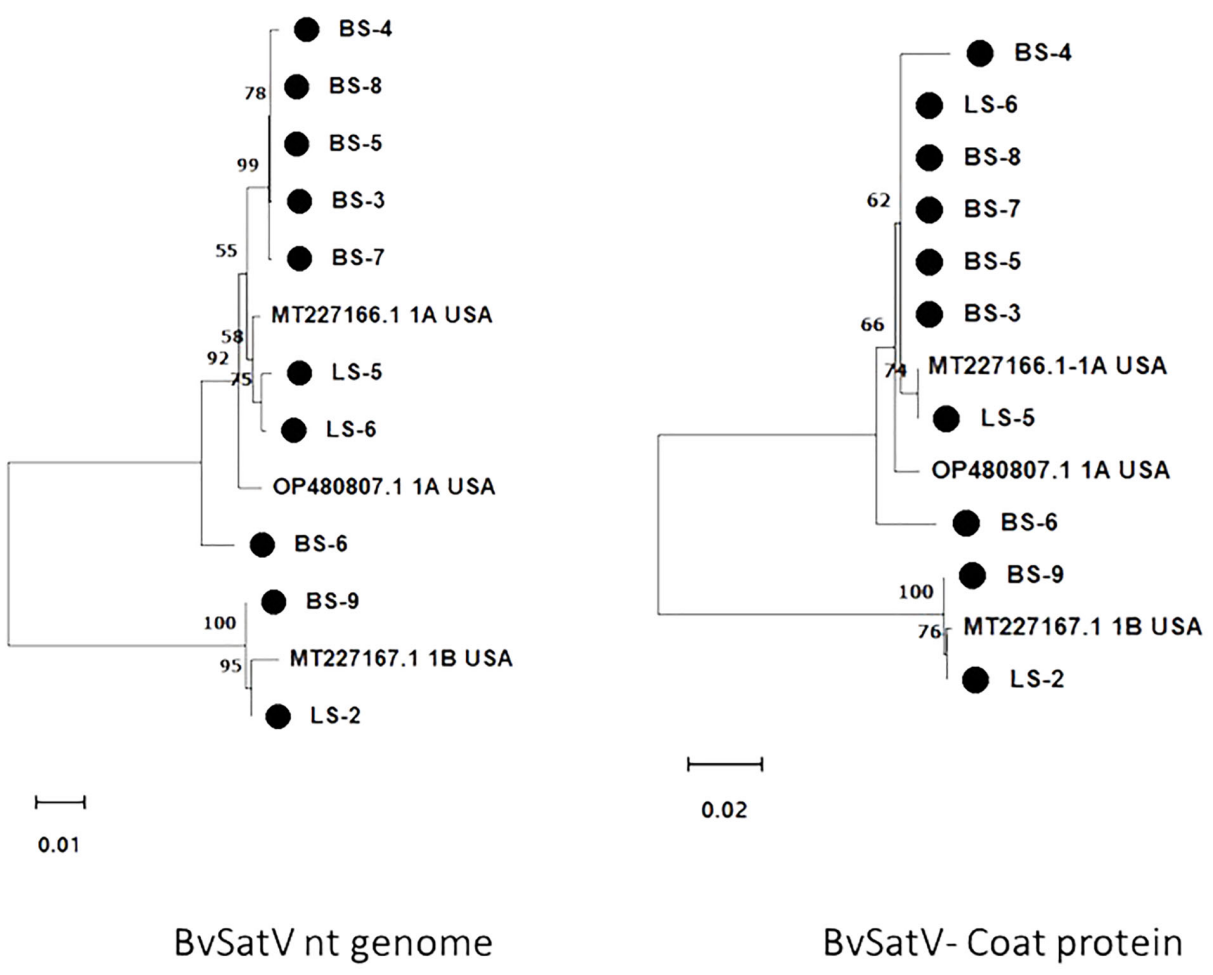
Maximum likelihood phylogram constructed with 1000 bootstrap replications for BvSatV nt genome and the deduced amino acid (aa) sequence of coat protein with previously reported isolates. Nucleotide sequences were trimmed to equal lengths of 911 nt, and complete amino acid (215 aa) sequences of coat protein were used. Two clusters were formed in phylogram with two isoforms of BvSatV where one cluster represented isolates closely matching with BvSatV-1A isoform and the other cluster represented isolates matching with BvSatV-1B isolate. • Marks denote isolates from this study.

The RT-PCR products were confirmed by Sanger sequencing and BLASTn analysis of the obtained sequences revealed the presence of the corresponding viral target sequences (**Supplementary Table 4**). The nucleotide sequence identity for BNYVV was obtained as follows: BNYVV-RNA1 (99.56-99.85%), BNYVV-RNA2 (99.35-99.67%), BNYVV-RNA3 (99.36-100%), and BNYVV-RNA4 (99.81-100%). For BSBMV, the sequence identities are as follows: BSBMV-RNA1 (99.36-99.47%), BSBMV-RNA2 (98.87-99.09%), BSBMV-RNA3 (98.13-99.54%), and BSBMV-RNA4 (98.93-99.54%). Sequence identity for BSBV: BSBV-RNA1 (99.03-99.72%), BSBV-RNA2 (99.21-99.34%), and BSBV-RNA3 (98.78-99.84%). In the case of BvSatV, BLASTn search identified two isoforms, BvSatV-1A and BvSatV-1B, among the samples. A primer sequence conserved between the two variants of BvSatV was used to amplify both the isoforms of BvSatV. Sequencing of the RT-PCR amplicons obtained for BvSatV revealed that the BS-9 root sample from Idaho and the leaf sample LS-2 from Minnesota were found to match with BvSatV-1B with 98.06%-98.21% identity. The rest of the samples that were positive for BvSatV matched to BvSatV-1A with 98.62%-98.79% identity (**Figures 3**, **4**).

### 3.4 Genome characterization of *Erysiphe necator-*associated abispo virus

**Figure 5:**
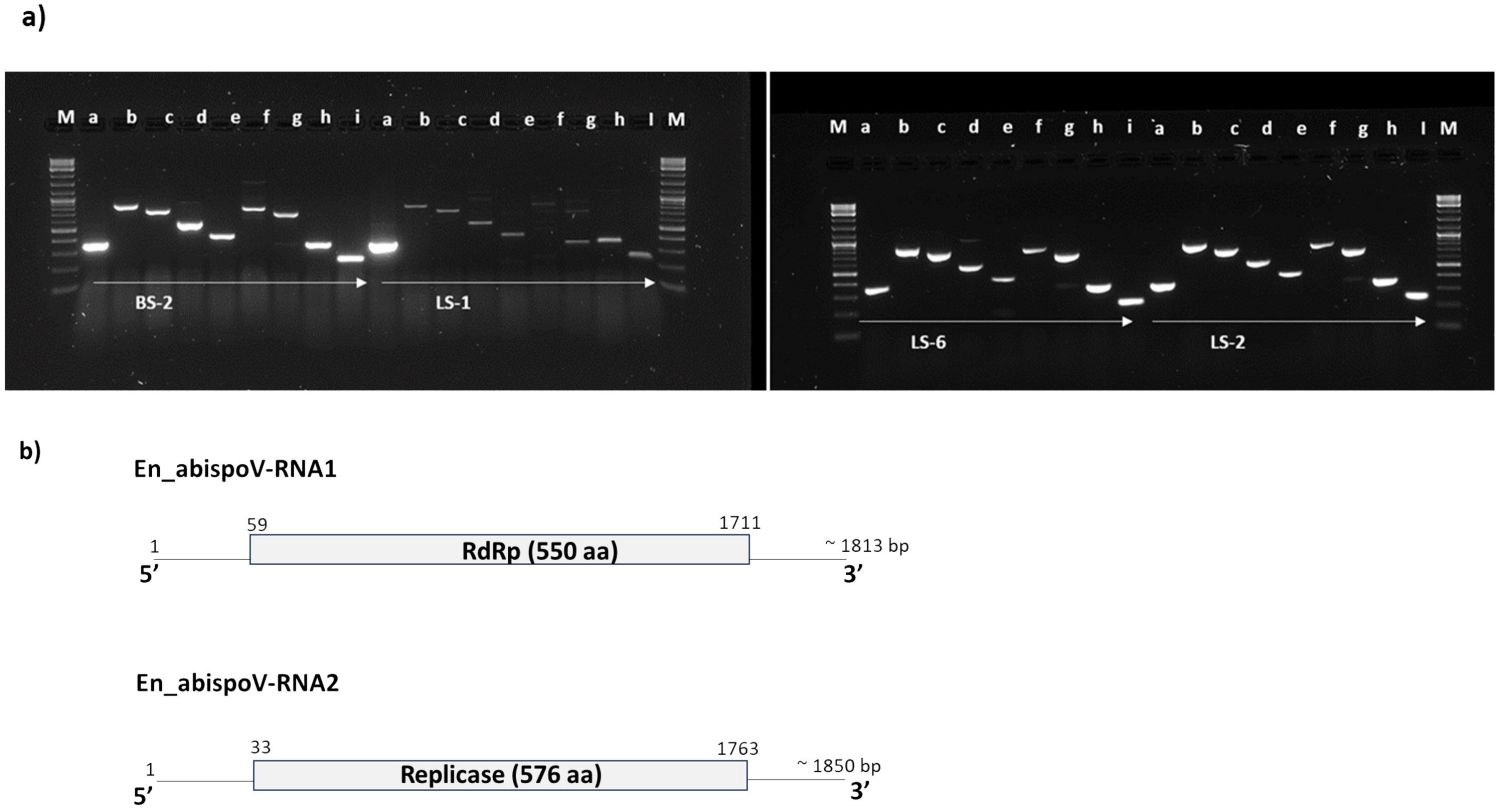
**(A)** Overlapping genome fragments of En_abispoV RNA1 and RNA2 were amplified in RT-PCR following primer walking strategies. Lane: M, 1 kb plus DNA ladder; a through e, overlapping genome fragments of RNA1 (a, 364 bp; b, 863 bp; c, 769 bp; D, 575 bp; and e, 439 bp); f through i, overlapping genome fragments of RNA2 (f, 819 bp,;g, 712 bp; h, 369bp; and i, 258 bp). Primer details are given in **Supplementary Table 1**. In LS-1, the target genome fragments e and f showed mild amplification and these amplicons were subjected to reamplification in RT-PCR reactions and the target regions obtained. **(B)** Graphical representation of the genome organization of En_abispoV and the RNA1 and RNA2 components. The numbers on the genome indicate the nucleotide positions of the genome and the deduced ORFs are indicated in open boxes with annotation.

In addition to RNASeq-aided extraction of the complete genome sequence of En_abispoV in 14 libraries, we used contiguous overlapping regions of RNA1 and RNA2 to validate the HTS results. Using RT-PCR and primer walking strategies, the near-complete sequences of En_abispoV RNA1 and RNA2 were obtained in one of the root samples (BS-2) and two leaf samples (LS-1 and LS-2) originating from Minnesota, and a leaf sample from Colorado (LS-6) (**Figure 5**). All the specific sizes of PCR amplicons were gel-purified and sequenced. Among the four samples, LS-1 showed very mild amplification for a few fragments of RNA1 and RNA2 where RT-PCR amplicons were used as templates to reamplify the regions. BLASTn analysis with overlapping sequences of RNA1 showed 94.12% to 97.01% identity with *Erysiphe necator-*associated abispo virus-8 (MN611695.1), and RNA 2 sequences showed 94.36% to 95.36% identity with *Erysiphe necator-*associated abispo virus-7 isolate (MN611694.1). The nearly complete genomes of RNA1 and RNA2 were constructed using the overlapping sequences obtained through RT-PCR and Sanger sequencing and the reconstructed sequences matched to the corresponding reference sequences. The near-complete genome of RNA1 and RNA2, compared in all four samples, showed a range of 1691 to 1704 nts for RNA1 and 1790 to 1799 nts for RNA2.

These RNA1 and RNA2 genome sequences that were obtained from all four samples harbored the full-length open-reading frame (ORF). The ORF finder from the NCBI showed a single open reading frame in RNA1 and RNA2 for all four samples that was 1653 bp and 1731 bp in size, respectively. CDD-NCBI, Pfam 35.0, and InterPro 91.0 from the EMBL-EBI identified a conserved domain in the ORF of RNA1, which was 550 amino acid residues in size and belonged to the ps-ssRNAv-RdRp-like superfamily (cl40470). The ORF in RNA2 had 576 amino acid residues and contained a Vmethyltransf superfamily (cl03298) conserved domain. RNA1 and RNA2 of all four samples were annotated using GATU and submitted to GenBank and accession numbers were obtained (PP747086 - PP747093).

### 3.5 Full genome assembly of viruses and phylogenetic analysis of BvSatV and En_abispoV

The complete and nearly complete genome sequences of the major sugarbeet-infecting viruses BNYVV, BSBMV, and BSBV were obtained from the HTS results. BNYVV was the most widespread and its genome sequence was extracted in seven root libraries wherein the assembled genome for RNA1 ranged from 6749 to 6698 nt, for RNA2 from 4576 to 4620 nt, for RNA3 from 1740 to 1780 nt, and for RNA4 from 1437 to 1543 nt. The BNYVV isolates obtained in this study showed 98.62% – 99.98% nucleotide sequence identities in RNA1 through RNA4 to the previously reported BNYVV U.S. isolate. The *de novo* assembled BSBMV genome sequences from two root libraries revealed the complete sequence of RNA1 (6656 to 6668 nt), RNA2 (4583 to 4594 nt), RNA3 (1697 to 1713 nt), and RNA4 (1702 to 1769 nt). Comparison of the BSBMV RNA sequences revealed 98.23%-99.88% identities to the U.S. isolate of BSBMV. The near-complete sequences of BSBV genome RNA1 (5818 to 5834 nt), RNA2 (3427 to 3453 nt), and RNA3 (3002 to 3006 nt) were obtained in four root libraries. Sequence comparison at the nucleotide level revealed 98.23%-99.88% similarities to the previously reported BSBV. GenBank accession numbers are provided for each of the viruses used in the analysis (**Supplementary Table 4**).

The genome sequence of BvSatV was assembled in seven roots and three leaf libraries using HTS data that ranged from 1118 to 1210 nt, and were submitted to GenBank, and accession numbers were obtained (PP739046 - PP739055). Phylogenetic analysis showed two clusters, each specific to isoform BvSatV-1A and BvSatV-1B, respectively. Among the two isoforms, BvSatV-1A was widespread and detected in eight libraries, and BvSatV-1B was found only in two libraries (LS-2 and BS-9). BvSatV-1A identified in this study revealed 97.58% – 99.28% nt identity with the previously reported BvSatV-1A strain MT227166.1, while the BvSatV-1B is closely related to the 1B isolate MT227167.1 with 99.32% - 99.45% nt identities. Overall, the isolates from the BvSatV-1A cluster shared 89.6% to 90.66% identity at genome sequence level; however, it shared slightly less homology at the amino acid sequence in the coat protein coding region with 84.18% to 85.58% to the isolates of BvSatV-1B cluster (**Supplementary Table 5**; **Figure 4**).

**Figure 6:**
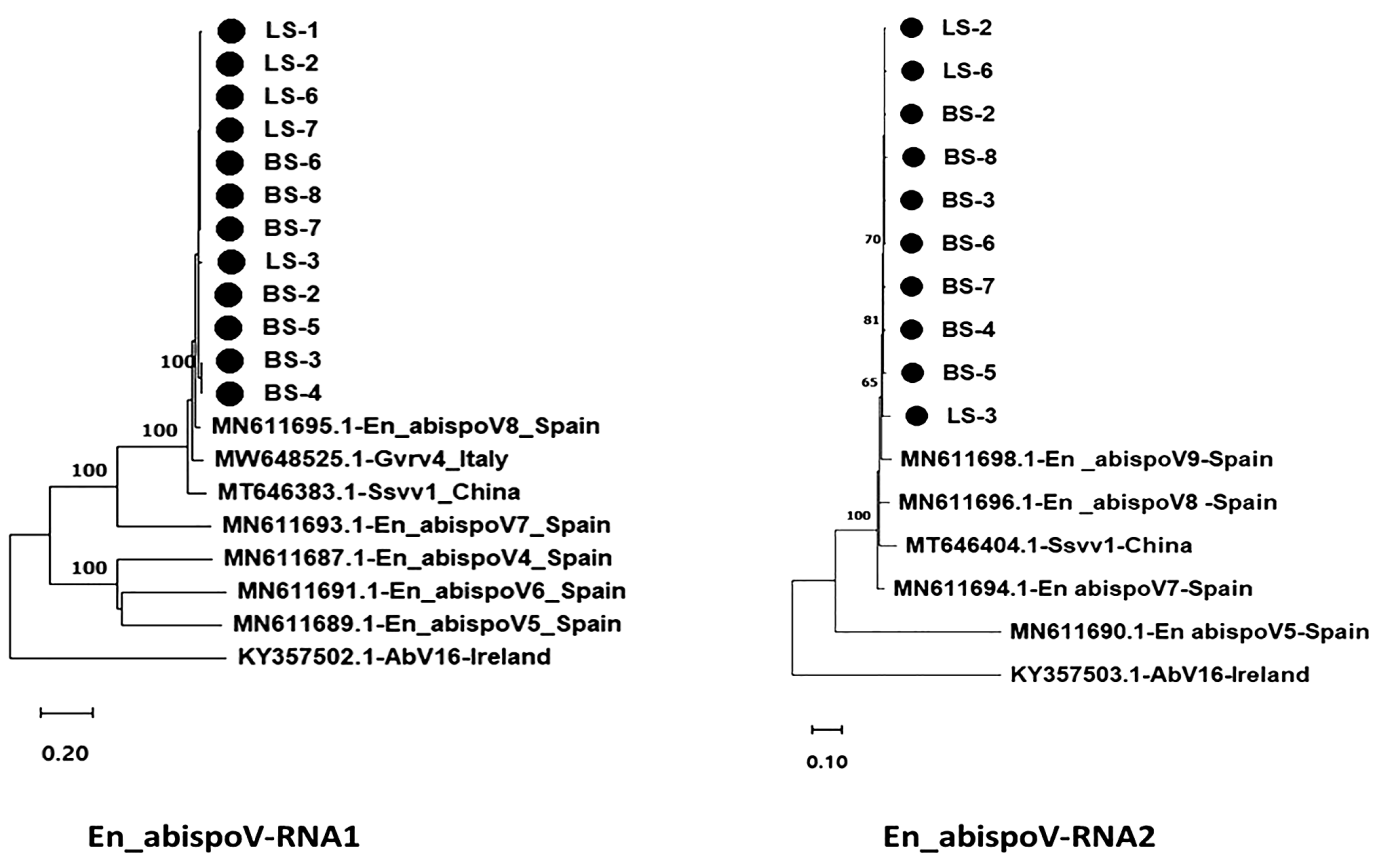
Maximum likelihood phylogram constructed with 1000 bootstrap replications for En_abispoV RNA1 and RNA 2 sequences with closely matched isolates obtained from GenBank. Nucleotide sequences are trimmed to equal lengths such as En_abispoV RNA1 with 1773 nt, and En_abispoV RNA2 with 1855 nt sequences. RNA1 isolates from sugar beet showed a close relationship with *Erysiphe necator-associated* abispo virus 8 isolate PMS7_214 (MN611695.1) from Spain followed by Grapevine-associated RNA virus 4 RNA-dependent RNA polymerase gene (MW648525.1) from Italy. In the case of RNA2, *Erysiphe necator*-associated abispo virus 8 isolate PMS7_153 (MN611696.1), *Erysiphe necator* associated abispo virus 9 isolate PMS8_99 (MN611698.1) and *Erysiphe necator* associated abispo virus 7 isolate PMS5_242 (MN611694.1) from Spain shared a close relationship with the sugar beet isolates. *Agaricus bisporus* virus-16 isolate AbV16-003 from Ireland shared a distant relationship with both the RNA1 and RNA2 sequences compared to all other isolates with less than 50% nt identities. • Marks denote isolates from this study.

Using HTS data, the En_abispoV genome sequence was assembled in 12 libraries. The size of RNA1 ranged from 1796 to 1813 nts, and the RNA2 range from 1572 to 1850 nts. The obtained sequences were submitted to GenBank, and obtained accession numbers (PP739024 - PP739045). Phylogenetic analysis of the En_abispoV isolates identified in this study showed 43.80 -97.18% nt identities with the RNA1 of other related isolates that are used in the phylogenetic analysis. However, the RNA2 appears highly diverse sharing 32.60 - 94.58% identities with other related isolates (**Figure 6**). Among them, *Erysiphe necator-*associated abispo virus 8 isolate PMS7_214 (MN611695.1) from Spain showed 95.59% to 97.18% identities at the nt level with RNA1 sequences of this study. Further, the amino acid sequences of RdRp in RNA1 shared 98.54% to 99.68% similarities with the same isolate. In the case of RNA2, *Erysiphe necator*-associated abispo virus 7 isolate PMS5_242 (MN611694.1), *Erysiphe necator-*associated abispo virus 9 isolate PMS8_99 (MN611698.1), and *Erysiphe necator-*associated abispo virus 8 isolate PMS7_153 (MN611696.1) from Spain shared a close relationship with 91.86% to 94.58% nt identities and 98.84% to 99.82% similarities at the amino acids of replicase in RNA 2 (**Figure 6**; **Supplementary Table 5**). It is important to note that the RdRp in En_abispoV-RNA1 and the replicase in En_abispoV-RNA2 shared less than 35% aa similarity among the isolates identified in this study. *Sclerotinia sclerotiorum* virga-like virus 1 RNA1 (MT646383.1) and RNA2 (MT646404.1) shared 89.6% to 91.3% nt similarities with En_abispoV RNA1 and 2, respectively. *Agaricus bisporus* virus-16 isolate shared the least similarities with En_abispoV RNA1 and RNA2 with 43.8%-48.1% and 32.6%-45.0% nt identities, respectively (**Figure 6**).

## 4 Discussion

Meta-transcriptomic sequencing has dramatically increased our current knowledge of the natural diversity of viruses in several crops (Roossinck et al., 2015). The purpose of this study was to identify naturally inhabiting viruses in the roots and leaves of field-grown sugarbeet plants using a meta-transcriptomic approach. The sugarbeet samples analyzed in the study had hairy root symptoms resembling rhizomania or had leaf samples exhibiting a range of symptoms including leaf curling and yellowing that are reminiscent of virus yellows and curly top, known foliar viral diseases of sugarbeet. RNA-seq analyses revealed the occurrence and distribution of seven major sugarbeet infecting viruses that included BNYVV, BSBV, BSBMV, and BCTV; the recently discovered BvSatV isoforms; BvANV-1; and a new En_abispoV identified in this study.

Among the seven viruses, BNYVV was identified in all nine root libraries irrespective of the origin of the samples. However, the coverage across all four RNAs was low in the samples BS-7 and BS-8, which correlated to the mild rhizomania-like symptoms observed in these beet samples. The other soil-borne viruses that co-exist with BNYVV, the BSBMV and BSBV, were also identified in samples in this study. BSBMV was detected in six root libraries and one of the leaf libraries, and all these samples originated from Minnesota and North Dakota sugarbeet growing fields. However, BSBMV was not detected in the root or leaf samples that were obtained from California, Colorado, and Idaho. The other soil-borne virus, BSBV, was primarily identified with high coverage in four root libraries, and with low coverage in BS-4, BS-7, and in the three leaf samples, revealing BSBV incidence in Idaho and Colorado, in addition to Minnesota and North Dakota. No sequencing reads representing BSBV were detected in the sample from California in this analysis.

BvSatV is a satellite virus that was first originally discovered from North Dakota and Minnesota sugarbeet production fields (Weiland et al., 2020). In this study, BvSat-1A and BvSat-1B, the two isoforms, were identified in Colorado, and Idaho, the two new locations of the western sugarbeet growing areas in the U.S. in addition to North Dakota and Minnesota. In fact, BvSatV was the second most dominant known virus as it was detected in 13 libraries from root and leaf samples that originated from Minnesota, North Dakota, Colorado, and Idaho, indicating the widespread occurrence of BvSatV in the U.S. Of note, recently, BSBV was found to serve as the helper virus for BvSatV replication (Weiland et al.,2024). Consistent with this, a correlation between the co-existence of BSBV and BvSatV was observed, supporting that BSBV is most likely a helper virus for BvSatV. However, it is also worth noting that in two leaf samples (LS-4 and LS-9), BvSatV was detected without the presence of BSBV; thus, the possible involvement of other viruses to serve as an alternative helper virus can not been excluded. HTS reads mapping to BVQ, and another soil-borne virus, *Beet black scorch virus* (*Betanecrovirus*) were not detected in any of the tested samples. Among the known foliar viruses, BCTV is the causal agent of curly top disease (Strausbaugh et al., 2017), and it was detected only in the sample from Idaho. Further, the viruses BChV, BWYV, and BYV that are associated with yellows disease (Hossain et al., 2021; Kozłowska-Makulska et al., 2009; Wintermantel, 2005) were undetectable in all the tested 18 libraries originating from across the U.S. Phylogenetic analysis revealed an overall low diversity among the BNYVV isolates identified in this study, and that these isolates showed high degree of similarity to the U.S. isolate of BNYVV in all the four RNA components (Weiland et al., 2020). The two isolates of BSBMV, a virus detected only in the U.S (Lee et al., 2001), and the two isolates from this study were randomly clustered with previously reported U. S. isolates. BSBV isolates of this study shared more than 98% homology for RNA1 to RNA3 with the previously reported U. S. (OP380963) and Brazilian (MH106715) isolates.

The newly identified En_abispoV was the most widespread virus sequence, next to BNYVV, detected among the identified viruses, and was represented in 14 samples that originated from Minnesota, Colorado, and North Dakota. Using HTS results, the nearly complete sequences of RNA1 and RNA2 of En_abispoV were assembled, which were confirmed using RT-PCR and Sanger sequencing. En_abispoV appears to be a new, as yet unclassified, virus that remains poorly characterized. The association of the virus with the fungus, *E. necator*, and the available virus-sequence information in GenBank comprise, to date, the sole documentation on this group of viruses. Taxonomically, these En_abispoV isolates are grouped under the “Unclassified Riboviria”, where unclassified new or novel virus genomes that encode RNA-dependent RNA Polymerase (RdRp) or the RNA-dependent DNA Polymerase (RdDp) are included. As the name indicates, the genome structure of En_abispoV RNA1 and RNA2 resemble members of a mycovirus category with *E. necator* as the potential host. Several mycovirus-related RNA viruses other than the En_abispoV have been detected in *E. necator* (Pandey et al., 2018). However, a BLASTn search with *de novo* assembled contigs with specific criteria that included being more than 350 bp length, > 80% nucleotide identity, and > 80% coverage resulted in no contigs closely matching with the genomic fragments of fungus *Erysiphe necator* in any of the libraries analyzed in this study. In addition to aligning to En_abispoV, the assembled contigs matched with *Sclerotinia sclerotiorum* virga-like virus 1 (MT646383.1) in BLASTn analysis with 90% to 92% sequence similarities. The necrotrophic fungus *S. sclerotiorum* causes sclerotinia head and stalk rot and is one of the major production constraints of sunflower (*Helianthus annuus* L.) in the U.S. *Erysiphe necator* or its related species that cause powdery mildew, is a common disease of grapevine worldwide (Qiu et al., 2015). It is tempting to speculate that there was an existence of *E. batae*, and that the En_abispoV was associated with *E. batae*. In addition, phylogenetic analysis of En-abispoV RdRp showed 98.9% amino acid similarity with Grapevine-associated RNA virus 4 (MW648525.1) found in *Vitis vinifera* from Italian isolate, En_abispoV isolate 8, which is grouped under ‘Unclassified Riboviria’. This study has identified several co-existing viruses including BNYVV, BSBMV, BSBV, BvSatV, and the newly characterized En_abispoV. In mixed infections, viruses can interact synergistically wherein the presence of one virus can enhance the replication and transmission of other viruses (Vanitharani et al., 2004). Consistent with this notion, BSBV was recently identified as the helper virus for BvSatV (Weiland et al., 2024). The identification of En_abispo virus may be due to the application of meta-transcriptomic analysis since mycoviruses often exist asymptomatically in their hosts. Hence, further research will be required to experimentally validate the interactions among co-existing viruses to elucidate the nature and mechanisms of these interactions.

The abispo virus terminology was introduced with a virus that was identified in *Agaricus bisporus*, the *Agaricus bisporus virus 16* (AbV16) with RNA1 containing the RdRP domain and RNA2 encoding a methyltransferase domain associated with brown cap mushroom disease (Deakin et al., 2017). The En_abispoV isolates of sugarbeet are unlikely to be related to AbV16 since these isolates shared less than 50% sequence identity with the RNA1 and RNA2 of the corresponding AbV16 components. Based on our study, we infer that the newly identified En_abispoV might have been transferred to sugarbeet from *E. necator* (syn. *Uncinula necator*) or *S. sclerotiorum*, or perhaps via soil carrying the fungi.

The meta-transcriptomic approach provides the opportunity to understand the occurrence of natural viromes under environmental conditions, which are underestimated when using specific practices for targeted sequencing approaches including culturing of pure cultures for pathogenic organisms (Roossinck et al., 2015). The agricultural practice of crop rotation provides an opportunity to dilute the plant-pathogen interactions, but it also offers new opportunities for novel adaptations. Evidence for the transfer of viruses between plants and fungi has been reported. For example, the acquisition of cucumber mosaic virus (CMV) by the phytopathogenic fungus *Rhizoctonia solani* from an infected plant as well as the ability of fungi to transmit the virus to the uninfected plants was reported (Andika et al., 2017). The replication of tobacco mosaic virus (TMV) was confirmed in phytopathogenic fungus *Colletotrichum falcatum* (Mascia et al., 2014). Similarly, *Magnaporthe oryzae*, the causal agent of the rice blast, was reported with virus infections from different families such as *Victorivirus*, *Partitivirus*, *Chrysovirus*, and *Tombusvirus* (Moriyama et al., 2018).

This study revealed the occurrence and distribution of sugarbeet-infecting viruses in the U.S. through a meta-transcriptomic approach followed by RT-PCR-based validation. Among the known sugarbeet-associated viruses, the geographical expansion of the BvSatV isoforms (BvSat-1A and BvSat-1B) was noticeable. Above all, we provide evidence for the identification of En_abispoV, a new virus naturally occurring in sugarbeet, which is prevalent across the country. In nature, virus adaptation to new hosts is influenced by holistic interactions among the host, insect vector, coexisting viruses, and environmental factors. The widespread occurrence of En-abispoV in sugarbeet needs further investigation to understand the host range and biology of the virus. These results set the baseline for further research to navigate towards understanding the biological significance of this virus for sugarbeet productivity.

## Data Availability

The datasets presented in this study can be found in online repositories. The names of the repository/repositories and accession number(s) can be found in the article/**Supplementary Material**.
