# Supplementary material for: Meta-transcriptomic analysis reveals the geographical expansion of known sugarbeet-infecting viruses and the occurrence of a novel virus in sugarbeet in the United States
Source: Front Plant Sci. 2024 Aug 30;15:1429402. doi: 10.3389/fpls.2024.1429402 (PMC11407286; doi:10.3389/fpls.2024.1429402)
Supplement: Supplementary file 1 [file Table1.docx]

Supplementary Material

**Supplementary Table 1** Details of primers used in RT-PCR for the diagnosis of sugarbeet infecting viruses and genome characterization of *Erysiphe necator*-associated abispo virus in this study.

| **Primer No** | **Target genome** | **Primer name** | **Primer sequence (5' to 3')** | **Target size** |
| --- | --- | --- | --- | --- |
| 1 | BNYVV-RNA1 | VR-98 | TCTGAGGGGACATCCTATTGCT |  |
| 2 | BNYVV-RNA1 | VR-106 | GGACTGCATAACATATTTCTCACG | 729 |
| 3 | BNYVV-RNA-2 | VR-273 | CCGAGTCCATCACTAAGGTTGCTA |  |
| 4 | BNYVV-RNA-2 | VR-274 | AGGAGGCCTGTGGCATGGCCCTGA | 628 |
| 5 | BNYVV-RNA-3 | VR-277 | AGTCCGAACTTACAGTGTGGGTTC |  |
| 6 | BNYVV-RNA-3 | VR-278 | CTGTGGAACCCACGATGCCGCCGT | 504 |
| 7 | BNYVV-RNA-4 | VR-281 | CTGTCTGATGAGCTATGTGAAGCT |  |
| 8 | BNYVV-RNA-4 | VR-282 | CTTGTGAACGAGCCCGTTAATAGT | 567 |
| 9 | BSBV-RNA1 | VR-261 | CTGAACGTGGTACTCATAGTATGGCTGC |  |
| 10 | BSBV-RNA1 | VR-262 | CTGTCATAGTAAAGTACATCACGT | 772 |
| 11 | BSBV-RNA-2 | VR-285 | TCGTCCGTAGACTCCGCCACGAAC |  |
| 12 | BSBV-RNA-2 | VR-286 | CCGAACTCTCAATGTTAGCACTGG | 922 |
| 13 | BSBV-RNA-3 | VR-287 | GCCGGTCGCTTGTGATCAATTA |  |
| 14 | BSBV-RNA-3 | VR-288 | CAAGTTGTCTCTTACCCGCGTATC | 704 |
| 15 | BSBMV-RNA1 | VR-427 | GCTCCGGCTGATACTTTGAG |  |
| 16 | BSBMV-RNA1 | VR-428 | GTTGGCAACAAACTCCACCT | 828 |
| 17 | BSBMV-RNA2 | VR-433 | CGAGATCACTCGCGTTCATA |  |
| 18 | BSBMV-RNA2 | VR-434 | CTCAGGAGAGGACCAGCAAC | 912 |
| 19 | BSBMV-RNA3 | VR-439 | ATGTTATCCGCTTCCTGTCG |  |
| 20 | BSBMV-RNA3 | VR-440 | TTCGGACCCACACTGTCATA | 738 |
| 21 | BSBMV-RNA4 | VR-445 | GTTGTGCAATATCGGTGTGC |  |
| 22 | BSBMV-RNA4 | VR-446 | TTCCAAGGATCACTCGGTTC | 962 |
| 23 | BvSatV | VR-485 | GGCGAAATGATCAGCCGTCAAC |  |
| 24 | BvSatV | VR-486 | GACGACATGTCAAGGATTCG | 706 |
| 25 | BvANV-1 | VR-477 | GAGACGCCAACGTACAGTCA |  |
| 26 | BvANV-1 | VR-478 | TAGCGCAACAACTGGCGCTGT | 644 |
| 27 | En_abispoV-RNA1 | VR-491 | CCCGATGAGGACATTCAAGT |  |
| 28 | En_abispoV-RNA1 | VR-492 | CTCGGCATGCTTATGACAGA | 773 |
| 29 | En_abispoV-RNA1 | VR-491 | CCCGATGAGGACATTCAAGT |  |
| 30 | En_abispoV-RNA1 | VR-498 | CATCGGATCAAACTTTGCCATCC | 364 |
| 31 | En_abispoV-RNA1 | VR-501 | GAGACGTTGACCGCTTACAAGCAG |  |
| 32 | En_abispoV-RNA1 | VR-502 | TTCCAGGTCACTCAACCCGAGGT | 863 |
| 33 | En_abispoV-RNA1 | VR-503 | GGCACCACTTTCACCACTTGCCGCA |  |
| 34 | En_abispoV-RNA1 | VR-504 | TCAAGCCCTTCAGACCACTCTGAG | 769 |
| 35 | En_abispoV-RNA1 | VR-331 | TTGGGGTTTGCGGGTCA |  |
| 36 | En_abispoV-RNA1 | VR-333 | GGCGATGCAACTTTCCTAT | 575 |
| 37 | En_abispoV-RNA1 | VR-334 | CTTCGCCTTGAGCACCATTT |  |
| 38 | En_abispoV-RNA1 | VR-335 | CAAGCAGACCGACACAACTC | 439 |
| 39 | En_abispoV-RNA2 | VR-505 | CGGTAAGGCAAAATGTTCGT |  |
| 40 | En_abispoV-RNA2 | VR-506 | CTTGACGTTCACCTGCTTGA | 766 |
| 41 | En_abispoV-RNA2 | VR-509 | TGGACAACCCACATCAGAGA |  |
| 42 | En_abispoV-RNA2 | VR-510 | ATGTCGGAAAGTTGCTGAGCGT | 712 |
| 43 | En_abispoV-RNA2 | VR-511 | TCAGACACCTCATCTGTCACGGAC |  |
| 44 | En_abispoV-RNA2 | VR-512 | AATGAAACCATCGACGACTGCACC | 819 |
| 45 | En_abispoV-RNA2 | VR-513 | GCAAGATCAAGGCAAACTCACTCAT |  |
| 46 | En_abispoV-RNA2 | VR-514 | AATGGTCCACTGGCATCAT | 369 |
| 47 | En_abispoV-RNA2 | VR-515 | CAGACGTATGTCACTACCTTGGTCG |  |
| 48 | En_abispoV-RNA2 | VR-516 | GTGAGGCGTCCAGATTTGTTGTA | 258 |

**Supplementary Table 2.** The total number of contigs obtained for each RNA segment for major known and newly identified viruses in sugarbeet samples.

| BNYVV (>150 bp) | BS-1 | BS-2 | BS-3 | BS-4 | BS-5 | BS-6 | BS-7 | BS-8 | BS-9 | LS-2 | LS-5 | LS-6 | LS-7 | LS-8 |
| --- | --- | --- | --- | --- | --- | --- | --- | --- | --- | --- | --- | --- | --- | --- |
| RNA1 contigs | 7 | 2396 | 2039 | 1 | 833 | 605 | 3 | 3 | 42 | 0 | 2 | 4 | 2 | 0 |
| RNA2 contigs | 8 | 3540 | 2356 | 2 | 1685 | 1196 | 6 | 3 | 250 | 1 | 1 | 4 | 0 | 1 |
| RNA3 contigs | 39 | 5082 | 4666 | 1 | 2787 | 2702 | 1 | 2 | 1138 | 1 | 0 | 3 | 3 | 1 |
| RNA4 contigs | 93 | 2901 | 3034 | 1 | 1926 | 1957 | 2 | 2 | 445 | 0 | 0 | 2 | 0 | 1 |
|  |  |  |  |  |  |  |  |  |  |  |  |  |  |  |
| BSBMV (> 150 bp) | **BS-2** | **BS-3** | **BS-4** | **BS-5** | **BS-6** | **BS-7** | **LS-2** |  |  |  |  |  |  |  |
| RNA1 contigs | 0 | 1 | 0 | 5 | 1 | 1 | 0 |  |  |  |  |  |  |  |
| RNA2 contigs | 1 | 1 | 1 | 33 | 0 | 0 | 2 |  |  |  |  |  |  |  |
| RNA3 contigs | 1 | 59 | 0 | 2 | 1 | 0 | 0 |  |  |  |  |  |  |  |
| RNA4 v | 0 | 1 | 0 | 2 | 0 | 0 | 0 |  |  |  |  |  |  |  |
|  |  |  |  |  |  |  |  |  |  |  |  |  |  |  |
| BSBV (> 150 bp) | **BS-2** | **BS-4** | **BS-5** | **BS-6** | **BS-7** | **BS-9** | **LS-2** | **LS-5** | **LS-6** |  |  |  |  |  |
| RNA1 contigs | 56 | 1 | 411 | 102 | 0 | 2007 | 0 | 1 | 0 |  |  |  |  |  |
| RNA2 contigs | 8 | 2 | 465 | 73 | 2 | 1239 | 0 | 0 | 0 |  |  |  |  |  |
| RNA3 contigs | 32 | 0 | 966 | 114 | 3 | 2288 | 3 | 0 | 1 |  |  |  |  |  |
|  |  |  |  |  |  |  |  |  |  |  |  |  |  |  |
| En_abispoV  (> 150 bp) | **BS-2** | **BS-3** | **BS-4** | **BS-5** | **BS-6** | **BS-7** | **BS-8** | **LS-1** | **LS-2** | **LS-4** | **LS-3** | **LS-6** | **LS-7** | **LS-8** |
| RNA1 contigs | 43 | 5 | 23 | 65 | 2 | 66 | 81 | 2 | 21 | 1 | 4 | 7 | 4 | 2 |
| RNA2 contigs | 23 | 2 | 13 | 18 | 5 | 18 | 17 | 4 | 11 | 0 | 4 | 10 | 3 | 1 |

**Supplementary Table 4**. Validation of high-throughput sequencing (HTS)-identified viruses: Blastn analysis results show the nucleotide percentage identities of RT-PCR amplified BNYVV, BSBMV, BSBV, and BvSatV genome sequences within the samples.

| **Virus name** | **GenBank Accession number** | **Description** | **Nucleotide percentage (%) identity** |
| --- | --- | --- | --- |
| BNYVV-RNA1 | MT227164.1 | Beet necrotic yellow vein virus strain USA2020 segment RNA1, complete sequence | 99.56 - 99.85 |
| BNYVV-RNA2 | MT227165.1 | Beet necrotic yellow vein virus strain USA2020 segment RNA2, complete sequence | 99.35-99.67 |
| BNYVV-RNA3 | MT372841.1 | Beet necrotic yellow vein virus isolate S6 p25 protein (p25) gene, complete cds | 99.36-100 |
| BNYVV-RNA4 | MT372842.1 | Beet necrotic yellow vein virus isolate S6 p31 protein (p31) gene, complete cds | 99.81-100 |
| BSBMV-RNA1 | OQ335848.1 | Beet soil-borne mosaic virus isolate DSMZ PV-1035 segment RNA1, complete sequence | 99.36-99.47 |
| BSBMV-RNA2 | OQ335849.1 | Beet soil-borne mosaic virus isolate DSMZ PV-1035 segment RNA2, complete sequence | 98.87-99.09 |
| BSBMV-RNA3 | OQ335850.1 | Beet soil-borne mosaic virus isolate DSMZ PV-1035 segment RNA3, complete sequence | 98.13-99.54 |
| BSBMV-RNA4 | OQ335851.1 | Beet soil-borne mosaic virus isolate DSMZ PV-1035 segment RNA4, complete sequence | 98.93-99.54 |
| BSBV-RNA1 | OP380962.1 | Beet soil-borne virus isolate BSBV-US-RNA1 segment RNA1, complete sequence | 99.03-99.72 |
| BSBV-RNA2 | OP380963.1 | Beet soil-borne virus isolate BSBV-US-RNA2 segment RNA2, complete sequence | 99.21-99.34 |
| BSBV-RNA3 | OP380964.1 | Beet soil-borne virus isolate BSBV-US-RNA3 segment RNA3, complete sequence | 98.78-99.84 |
| BvSatV-1A | MT227166.1 | Beta vulgaris satellite virus 1A strain USA2020, complete sequence | 97.58-99.28 |
| BvSatV-1B | MT227167.1 | Beta vulgaris satellite virus 1B strain USA-ND, complete sequence | 99.32-99.45 |

**Supplementary Table 5**. Nucleotide identity and amino acid similarity percentage values were compared among the isolates of this study and previously published related isolates from different geographical regions based on the phylogenetic analysis for BvSatV and En_abispoV.

| **Virus** | **Length of the trimmed genome used** | **Nucleotide (nt) identity % / amino acid (aa) similarity (%)** | |
| --- | --- | --- | --- |
|  |  | **In between the isolates from this study** | **With closely matched isolates** |
| **BvSatV** | | | |
| BvSatV-1A full genome (nt) | 911 | 97.58-100 | 97.58-99.28 |
| BvSatV-1A coat protein (aa) | 215 | 96.2-100 | 96.74-100 |
| BvSatV-1B full genome (nt) | 911 | 99.68-99.74 | 99.32-99.45 |
| BvSatV-1B coat protein (aa) | 215 | 100 | 100 |
| BvSatV-1A vs 1B full genome (nt) | 911 | 89.60-90.66 | - |
| BvSatV-1A vs 1B coat protein (aa) | 215 | 84.18-85.58 | - |
| **En_abispoV- RNA1** | | | |
| Full genome (nt) | 1773 | 96.87-100 | 48.63-97.18 |
| RdRp (aa) | 550 | 99.17-100 | 54.67-99.6 |
| **En_abispoV- RNA2** | | | |
| full genome (nt) | 1855 | 94.32-98.52 | 17.7-94.58 |
| Replicase (aa) | 576 | 99.07-100 | 42.91-99.82 |
