# Supplementary material for: Meta-transcriptomic analysis reveals the geographical expansion of known sugarbeet-infecting viruses and the occurrence of a novel virus in sugarbeet in the United States
Source: Front Plant Sci. 2024 Aug 30;15:1429402. doi: 10.3389/fpls.2024.1429402 (PMC11407286; doi:10.3389/fpls.2024.1429402)
Supplement: Supplementary file 2 [file Table2.pdf]

Supplementary Table 3. Blastn analysis results of all the consensus contig sequences obtained for major viruses from each library.

Library Name	Virus name*	Consensus contig length (nt)	Best match; GenBank accession number	Best match; Description	Query Coverage %	Expect value	Percent Identity (%)	GenBank accession length (nt)	Blastn Max Score	Blastn Total Score
BS-1	BNYVV-RNA1	6733	MT227164.1	Beet necrotic yellow vein virus strain USA2020 segment RNA1, complete sequence	100.00	0	99.90	6747	12395	12395
BS-2	BNYVV-RNA1	6749	MT227164.1	Beet necrotic yellow vein virus strain USA2020 segment RNA1, complete sequence	99.00	0	99.93	6747	12414	12414
BS-3	BNYVV-RNA1	6737	MT227164.1	Beet necrotic yellow vein virus strain USA2020 segment RNA1, complete sequence	99.00	0	99.94	6747	12417	12417
BS-4	BNYVV-RNA1	6698	MT227164.1	Beet necrotic yellow vein virus strain USA2020 segment RNA1, complete sequence	99.00	0	99.93	6747	12340	12340
BS-5	BNYVV-RNA1	6743	MT227164.1	Beet necrotic yellow vein virus strain USA2020 segment RNA1, complete sequence	99.00	0	99.88	6747	12401	12401
BS-6	BNYVV-RNA1	6729	MT227164.1	Beet necrotic yellow vein virus strain USA2020 segment RNA1, complete sequence	99.00	0	99.93	6747	12397	12397
BS-7	BNYVV-RNA1	1786	EU330450.1	Beet necrotic yellow vein virus strain S8 segment RNA 1 replication-associated protein gene, complete cds	100.00	0	99.94	6684	3293	3293
BS-8	BNYVV-RNA1	3085	MT227164.1	Beet necrotic yellow vein virus strain USA2020 segment RNA1, complete sequence	100.00	0	100.00	6747	4213	5697
BS-9	BNYVV-RNA1	6732	MT227164.1	Beet necrotic yellow vein virus strain USA2020 segment RNA1, complete sequence	100.00	0	99.93	6747	12405	12405
LS-5	BNYVV-RNA1	801	MH106726.1	Beet necrotic yellow vein virus isolate Brazilian segment RNA1, complete sequence	100.00	0	99.88	6743	1474	1474
LS-6	BNYVV-RNA1	2058	MT227164.1	Beet necrotic yellow vein virus strain USA2020 segment RNA1, complete sequence	100.00	0	99.95	6747	3795	3795
LS-7	BNYVV-RNA1	559	MH106726.1	Beet necrotic yellow vein virus isolate Brazilian segment RNA1, complete sequence	100.00	0	100.00	6743	1033	1033
LS-8	BNYVV-RNA1	342	MH106726.1	Beet necrotic yellow vein virus isolate Brazilian segment RNA1, complete sequence	100.00	2.00E-176	100.00	6743	632	632
BS-1	BNYVV-RNA2	4576	MT227165.1	Beet necrotic yellow vein virus strain USA2020 segment RNA2, complete sequence	99.00	0	99.87	4610	8416	8416
BS-2	BNYVV-RNA2	4620	MT227165.1	Beet necrotic yellow vein virus strain USA2020 segment RNA2, complete sequence	99.00	0	99.96	4610	8475	8475
BS-3	BNYVV-RNA2	4620	MT227165.1	Beet necrotic yellow vein virus strain USA2020 segment RNA2, complete sequence	99.00	0	99.87	4610	8453	8453
BS-4	BNYVV-RNA2	4583	MT227165.1	Beet necrotic yellow vein virus strain USA2020 segment RNA2, complete sequence	100.00	0	99.98	4610	8458	8458
BS-5	BNYVV-RNA2	4606	MT227165.1	Beet necrotic yellow vein virus strain USA2020 segment RNA2, complete sequence	99.00	0	99.91	4610	8479	8479
BS-6	BNYVV-RNA2	4595	MT227165.1	Beet necrotic yellow vein virus strain USA2020 segment RNA2, complete sequence	100.00	0	99.87	4610	8455	8455
BS-7	BNYVV-RNA2	2349	MT227165.1	Beet necrotic yellow vein virus strain USA2020 segment RNA2, complete sequence	100.00	0	99.91	4610	4327	4327
BS-8	BNYVV-RNA2	1825	MT227165.1	Beet necrotic yellow vein virus strain USA2020 segment RNA2, complete sequence	100.00	0	100.00	4610	3371	3371
BS-9	BNYVV-RNA2	4595	MT227165.1	Beet necrotic yellow vein virus strain USA2020 segment RNA2, complete sequence	100.00	0	99.70	4610	8418	8418
LS-2	BNYVV-RNA2	607	MH106727.1	Beet necrotic yellow vein virus isolate Brazilian segment RNA2, complete sequence	100.00	0	100.00	4609	1122	1122

LS-5	BNYVV-RNA2	1031	MT227165.1	Beet necrotic yellow vein virus strain USA2020 segment RNA2, complete sequence	100.00	0	99.81	4610	1893	1893
LS-6	BNYVV-RNA2	1648	MT227165.1	Beet necrotic yellow vein virus strain USA2020 segment RNA2, complete sequence	100.00	0	99.76	4610	3022	3022
LS-7	BNYVV-RNA2	600	MT227165.1	Beet necrotic yellow vein virus strain USA2020 segment RNA2, complete sequence	100.00	0	100.00	4610	1109	1109
LS-8	BNYVV-RNA2	434	MH106727.1	Beet necrotic yellow vein virus isolate Brazilian segment RNA2, complete sequence	100.00	0	100.00	4609	802	802
BS-1	BNYVV-RNA3	1740	MT372837.1	Beet necrotic yellow vein virus isolate S4 p25 protein (p25) gene, complete cds	99.00	0	99.60	1769	3173	3173
BS-2	BNYVV-RNA3	1758	MT372841.1	Beet necrotic yellow vein virus isolate S6 p25 protein (p25) gene, complete cds	99.00	0	100.00	1770	3217	3217
BS-3	BNYVV-RNA3	1758	MT372841.1	Beet necrotic yellow vein virus isolate S6 p25 protein (p25) gene, complete cds	99.00	0	100.00	1770	3230	3230
BS-4	BNYVV-RNA3	1742	MT372841.1	Beet necrotic yellow vein virus isolate S6 p25 protein (p25) gene, complete cds	100.00	0	100.00	1770	3217	3217
BS-5	BNYVV-RNA3	1777	MT372841.1	Beet necrotic yellow vein virus isolate S6 p25 protein (p25) gene, complete cds	99.00	0	100.00	1770	3269	3269
BS-6	BNYVV-RNA3	1780	MT372841.1	Beet necrotic yellow vein virus isolate S6 p25 protein (p25) gene, complete cds	99.00	0	99.94	1770	3260	3260
BS-7	BNYVV-RNA3	1261	MT372841.1	Beet necrotic yellow vein virus isolate S6 p25 protein (p25) gene, complete cds	100.00	0	99.92	1770	2324	2324
BS-8	BNYVV-RNA3	1631	MT372831.1	Beet necrotic yellow vein virus isolate S1 p25 protein (p25) gene, complete cds	100.00	0	99.88	1761	3001	3001
BS-9	BNYVV-RNA3	1764	KX665538.1	Beet necrotic yellow vein virus isolate PV0467/Yu2 segment RNA3, complete sequence	100.00	0	99.72	1775	3230	3230
LS-2	BNYVV-RNA3	285	MK241668.1	Beet necrotic yellow vein virus clone 11_1 P25 gene, complete cds	100.00	7.00E-145	100.00	660	527	527
LS-5	BNYVV-RNA3	319	MK241668.1	Beet necrotic yellow vein virus clone 11_1 P25 gene, complete cds	100.00	1.00E-163	100.00	660	590	590
LS-6	BNYVV-RNA3	1391	MT372841.1	Beet necrotic yellow vein virus isolate S6 p25 protein (p25) gene, complete cds	100.00	0	99.86	1770	2558	2558
LS-7	BNYVV-RNA3	688	MT372839.1	Beet necrotic yellow vein virus isolate S5 p25 protein (p25) gene, complete cds	100.00	0	99.42	1763	1245	1245
LS-8	BNYVV-RNA3	375	MK241668.1	Beet necrotic yellow vein virus clone 11_1 P25 gene, complete cds	100.00	0	100.00	660	693	693
BS-1	BNYVV-RNA4	1499	MH106729.1	Beet necrotic yellow vein virus isolate Brazilian segment RNA4, complete sequence	98.00	0	99.52	1468	2675	2675
BS-2	BNYVV-RNA4	1539	MT372834.1	Beet necrotic yellow vein virus isolate S2 p31 protein (p31) gene, complete cds	97.00	0	99.80	1468	2697	2757
BS-3	BNYVV-RNA4	1528	MT372834.1	Beet necrotic yellow vein virus isolate S2 p31 protein (p31) gene, complete cds	96.00	0	99.80	1468	2697	2697
BS-4	BNYVV-RNA4	1437	MT372834.1	Beet necrotic yellow vein virus isolate S2 p31 protein (p31) gene, complete cds	100.00	0	100.00	1468	2654	2654
BS-5	BNYVV-RNA4	1543	MT372834.1	Beet necrotic yellow vein virus isolate S2 p31 protein (p31) gene, complete cds	95.00	0	99.80	1468	2699	2699
BS-6	BNYVV-RNA4	1506	MT372834.1	Beet necrotic yellow vein virus isolate S2 p31 protein (p31) gene, complete cds	97.00	0	99.86	1468	2702	2702
BS-7	BNYVV-RNA4	1391	MT372834.1	Beet necrotic yellow vein virus isolate S2 p31 protein (p31) gene, complete cds	100.00	0	99.71	1468	2547	2547
BS-8	BNYVV-RNA4	730	MT372836.1	Beet necrotic yellow vein virus isolate S3 p31 protein (p31) gene, complete cds	100.00	0	100.00	1389	1349	1349
BS-9	BNYVV-RNA4	1455	MT372840.1	Beet necrotic yellow vein virus isolate S5 p31 protein (p31) gene, complete cds	99.00	0	99.86	1458	2673	2673
LS-6	BNYVV-RNA4	605	MT372842.1	Beet necrotic yellow vein virus isolate S6 p31 protein (p31) gene, complete cds	100.00	0	99.83	1349	1112	1112
LS-7	BNYVV-RNA4	301	MT372842.1	Beet necrotic yellow vein virus isolate S6 p31 protein (p31) gene, complete cds	100.00	5.00E-152	99.67	1349	551	551
LS-8	BNYVV-RNA4	248	MT372842.1	Beet necrotic yellow vein virus isolate S6 p31 protein (p31) gene, complete cds	99.00	3.00E-123	100.00	1349	455	455
BS-2	BSBMV-RNA1	995	OQ335848.1	Beet soil-borne mosaic virus isolate DSMZ PV-1035 segment RNA1, complete sequence	100.00	0	99.90	6679	1832	1832
BS-3	BSBMV-RNA1	6656	OQ335848.1	Beet soil-borne mosaic virus isolate DSMZ PV-1035 segment RNA1, complete sequence	99.00	0	99.94	6679	12268	12268
BS-4	BSBMV-RNA1	218	OQ335848.1	Beet soil-borne mosaic virus isolate DSMZ PV-1035 segment RNA1, complete sequence	100.00	1.00E-107	100.00	6679	403	403

BS-5	BSBMV-RNA1	6668	OQ335848.1	Beet soil-borne mosaic virus isolate DSMZ PV-1035 segment RNA1, complete sequence	100.00	0	99.93	6679	12286	12286
BS-6	BSBMV-RNA1	1297	OQ335848.1	Beet soil-borne mosaic virus isolate DSMZ PV-1035 segment RNA1, complete sequence	100.00	0	99.92	6679	2390	2390
BS-7	BSBMV-RNA1	273	KX352033.1	Beet soil-borne mosaic virus isolate BSBMV-CA segment RNA1, complete sequence	100.00	3.00E-138	100.00	6674	505	505
LS-2	BSBMV-RNA1	320	OQ335848.1	Beet soil-borne mosaic virus isolate DSMZ PV-1035 segment RNA1, complete sequence	100.00	1.00E-162	99.69	6679	586	586
BS-2	BSBMV-RNA2	1184	NC_039225.1	Beet soil-borne mosaic virus isolate MRM06 segment RNA2, complete sequence	100.00	0	99.41	4615	2148	2148
BS-3	BSBMV-RNA2	4583	NC_039225.1	Beet soil-borne mosaic virus isolate MRM06 segment RNA2, complete sequence	100.00	0	99.43	4615	8320	8320
BS-4	BSBMV-RNA2	324	NC_039225.1	Beet soil-borne mosaic virus isolate MRM06 segment RNA2, complete sequence	100.00	8.00E-165	99.69	4615	593	593
BS-5	BSBMV-RNA2	4594	NC_039225.1	Beet soil-borne mosaic virus isolate MRM06 segment RNA2, complete sequence	99.00	0	99.26	4615	8285	8285
BS-6	BSBMV-RNA2	1145	NC_039225.1	Beet soil-borne mosaic virus isolate MRM06 segment RNA2, complete sequence	100.00	0	99.21	4615	2065	2065
BS-7	BSBMV-RNA2	509	NC_039225.1	Beet soil-borne mosaic virus isolate MRM06 segment RNA2, complete sequence	100.00	0	99.80	4615	935	935
LS-2	BSBMV-RNA2	1017	NC_039225.1	Beet soil-borne mosaic virus isolate MRM06 segment RNA2, complete sequence	100.00	0	99.41	4615	1845	1845
BS-2	BSBMV-RNA3	503	KX352171.1	Beet soil-borne mosaic virus isolate BSBMV-CA segment RNA3, complete sequence	100.00	0	99.80	1720	924	924
BS-3	BSBMV-RNA3	1713	KX352171.1	Beet soil-borne mosaic virus isolate BSBMV-CA segment RNA3, complete sequence	99.00	0	99.88	1720	3144	3144
BS-5	BSBMV-RNA3	1697	KX352171.1	Beet soil-borne mosaic virus isolate BSBMV-CA segment RNA3, complete sequence	100.00	0	98.65	1720	3013	3013
BS-6	BSBMV-RNA3	821	KX352171.1	Beet soil-borne mosaic virus isolate BSBMV-CA segment RNA3, complete sequence	100.00	0	98.78	1720	1461	1461
BS-3	BSBMV-RNA4	1769	OQ335851.1	Beet soil-borne mosaic virus isolate DSMZ PV-1035 segment RNA4, complete sequence	97.00	0	99.83	1730	3153	3153
BS-5	BSBMV-RNA4	1702	OQ335851.1	Beet soil-borne mosaic virus isolate DSMZ PV-1035 segment RNA4, complete sequence	99.00	0	99.88	1730	3121	3121
					0.00		0.00			
BS-2	BSBV-RNA1	5819	OP380962.1	Beet soil-borne virus isolate BSBV-US-RNA1 segment RNA1, complete sequence	100.00	0	99.47	5837	10575	10575
BS-4	BSBV-RNA1	1170	OP380962.1	Beet soil-borne virus isolate BSBV-US-RNA1 segment RNA1, complete sequence	100.00	0	99.91	5837	2156	2156
BS-5	BSBV-RNA1	5834	OP380962.1	Beet soil-borne virus isolate BSBV-US-RNA1 segment RNA1, complete sequence	99.00	0	99.49	5837	10611	10611
BS-6	BSBV-RNA1	5818	OP380962.1	Beet soil-borne virus isolate BSBV-US-RNA1 segment RNA1, complete sequence	100.00	0	99.59	5837	10615	10615
BS-7	BSBV-RNA1	306	MH106714.1	Beet soil-borne virus isolate Brazilian segment RNA1, complete sequence	100.00	8.00E-155	99.67	5819	560	560
BS-9	BSBV-RNA1	5819	OP380962.1	Beet soil-borne virus isolate BSBV-US-RNA1 segment RNA1, complete sequence	100.00	0	99.64	5837	10630	10630
LS-5	BSBV-RNA1	279	OP380962.1	Beet soil-borne virus isolate BSBV-US-RNA1 segment RNA1, complete sequence	100.00	2.00E-141	100.00	5837	516	516
LS-6	BSBV-RNA1	290	MH106714.1	Beet soil-borne virus isolate Brazilian segment RNA1, complete sequence	100.00	1.00E-147	100.00	5819	536	536
BS-2	BSBV-RNA2	3427	OP380963.1	Beet soil-borne virus isolate BSBV-US-RNA2 segment RNA2, complete sequence	100.00	0	99.50	3450	6235	6235
BS-4	BSBV-RNA2	1590	OP380963.1	Beet soil-borne virus isolate BSBV-US-RNA2 segment RNA2, complete sequence	100.00	0	99.50	3450	2892	2892
BS-5	BSBV-RNA2	3453	OP380963.1	Beet soil-borne virus isolate BSBV-US-RNA2 segment RNA2, complete sequence	99.00	0	99.36	3450	6250	6250

BS-6	BSBV-RNA2	3442	OP380963.1	Beet soil-borne virus isolate BSBV-US-RNA2 segment RNA2, complete sequence	100.00	0	99.48	3450	6257	6257
BS-7	BSBV-RNA2	524	MH106715.1	Beet soil-borne virus isolate Brazilian segment RNA2, complete sequence	100.00	0	99.43	3448	952	952
BS-9	BSBV-RNA2	3448	OP380963.1	Beet soil-borne virus isolate BSBV-US-RNA2 segment RNA2, complete sequence	100.00	0	99.74	3450	6320	6320
LS-2	BSBV-RNA2	294	MH106715.1	Beet soil-borne virus isolate Brazilian segment RNA2, complete sequence	100.00	2.00E-146	99.32	3448	532	532
BS-2	BSBV-RNA3	3002	OP380964.1	Beet soil-borne virus isolate BSBV-US-RNA3 segment RNA3, complete sequence	99.00	0	99.10	3005	5397	5397
BS-4	BSBV-RNA3	619	OP380964.1	Beet soil-borne virus isolate BSBV-US-RNA3 segment RNA3, complete sequence	100.00	0	99.84	3005	1138	1138
BS-5	BSBV-RNA3	3005	OP380964.1	Beet soil-borne virus isolate BSBV-US-RNA3 segment RNA3, complete sequence	99.00	0	99.23	3005	5419	5419
BS-6	BSBV-RNA3	3005	OP380964.1	Beet soil-borne virus isolate BSBV-US-RNA3 segment RNA3, complete sequence	99.00	0	99.37	3005	5443	5443
BS-7	BSBV-RNA3	1399	OP380964.1	Beet soil-borne virus isolate BSBV-US-RNA3 segment RNA3, complete sequence	100.00	0	99.07	3005	2512	2512
BS-9	BSBV-RNA3	3006	OP380964.1	Beet soil-borne virus isolate BSBV-US-RNA3 segment RNA3, complete sequence	99.00	0	99.27	3005	5424	5424
LS-2	BSBV-RNA3	1380	OP380964.1	Beet soil-borne virus isolate BSBV-US-RNA3 segment RNA3, complete sequence	100.00	0	99.13	3005	2483	2483
LS-5	BSBV-RNA3	563	OP380964.1	Beet soil-borne virus isolate BSBV-US-RNA3 segment RNA3, complete sequence	100.00	0	97.51	3005	963	963
LS-6	BSBV-RNA3	396	OP380964.1	Beet soil-borne virus isolate BSBV-US-RNA3 segment RNA3, complete sequence	100.00	0	99.24	3005	715	715
BS-2	BvSatV-1A	458	OP480807.1	Beta vulgaris satellite virus 1A isolate USA2020, complete sequence	100.00	0	94.98	1211	719	719
BS-3	BvSatV-1A	1202	OP480807.1	Beta vulgaris satellite virus 1A isolate USA2020, complete sequence	100.00	0	98.09	1211	2093	2093
BS-4	BvSatV-1A	1202	OP480807.1	Beta vulgaris satellite virus 1A isolate USA2020, complete sequence	100.00	0	98.00	1211	2087	2087
BS-5	BvSatV-1A	1210	OP480807.1	Beta vulgaris satellite virus 1A isolate USA2020, complete sequence	99.00	0	98.26	1211	2108	2108
BS-6	BvSatV-1A	1118	OP480807.1	Beta vulgaris satellite virus 1A isolate USA2020, complete sequence	100.00	0	96.51	1211	1849	1849
BS-7	BvSatV-1A	1180	OP480807.1	Beta vulgaris satellite virus 1A isolate USA2020, complete sequence	100.00	0	98.05	1211	2052	2052
BS-8	BvSatV-1A	1159	OP480807.1	Beta vulgaris satellite virus 1A isolate USA2020, complete sequence	100.00	0	98.19	1211	2025	2025
LS-4	BvSatV-1A	584	OP480807.1	Beta vulgaris satellite virus 1A isolate USA2020, complete sequence	100.00	0	98.12	1211	1018	1018
LS-5	BvSatV-1A	1204	OP480807.1	Beta vulgaris satellite virus 1A isolate USA2020, complete sequence	100.00	0	98.51	1211	2124	2124
LS-6	BvSatV-1A	1210	OP480807.1	Beta vulgaris satellite virus 1A isolate USA2020, complete sequence	100.00	0	98.68	1211	2146	2146
LS-9	BvSatV-1A	286	OP480807.1	Beta vulgaris satellite virus 1A isolate USA2020, complete sequence	100.00	2.00E-135	97.90	1211	496	496
BS-9	BvSatV-1B	1209	MT227167.1	Beta vulgaris satellite virus 1B strain USA-ND, complete sequence	75.00	0	99.41	905	1232	1634
LS-2	BvSatV-1B	1190	MT227167.1	Beta vulgaris satellite virus 1B strain USA-ND, complete sequence	74.00	0	99.26	905	1227	1608
BS2	En_abispoV-RNA1	1808	MN611695.1	Erysiphe necator associated abispo virus 8 isolate PMS7_214 segment RNA1, complete sequence	97.00	0	97.10	1758	2961	2961
BS3	En_abispoV-RNA1	1796	MN611695.1	Erysiphe necator associated abispo virus 8 isolate PMS7_214 segment RNA1, complete sequence	97.00	0	96.92	1758	2935	2935
BS4	En_abispoV-RNA1	1813	MN611695.1	Erysiphe necator associated abispo virus 8 isolate PMS7_214 segment RNA1, complete sequence	96.00	0	96.92	1758	2940	2940
BS5	En_abispoV-RNA1	1811	MN611695.1	Erysiphe necator associated abispo virus 8 isolate PMS7_214 segment RNA1, complete sequence	97.00	0	97.27	1758	2981	2981
BS6	En_abispoV-RNA1	1804	MN611695.1	Erysiphe necator associated abispo virus 8 isolate PMS7_214 segment RNA1, complete sequence	97.00	0	96.12	1758	2863	2863
BS7	En_abispoV-RNA1	1806	MN611695.1	Erysiphe necator associated abispo virus 8 isolate PMS7_214 segment RNA1, complete sequence	97.00	0	96.18	1758	2868	2868
BS8	En_abispoV-RNA1	1800	MN611695.1	Erysiphe necator associated abispo virus 8 isolate PMS7_214 segment RNA1, complete sequence	97.00	0	96.11	1758	2850	2850

LS1	En_abispoV-RNA1	1500	MN611695.1	Erysiphe necator associated abispo virus 8 isolate PMS7_214 segment RNA1, complete sequence	100.00	0	95.73	1758	2416	2416
LS2	En_abispoV-RNA1	1802	MN611697.1	Erysiphe necator associated abispo virus 9 isolate PMS8_32 segment RNA1, complete sequence	99.00	0	95.89	1849	2913	2913
LS3	En_abispoV-RNA1	1796	MN611695.1	Erysiphe necator associated abispo virus 8 isolate PMS7_214 segment RNA1, complete sequence	97.00	0	95.99	1758	2837	2837
LS4	En_abispoV-RNA1	736	MN611695.1	Erysiphe necator associated abispo virus 8 isolate PMS7_214 segment RNA1, complete sequence	100.00	0	95.52	1758	1177	1177
LS6	En_abispoV-RNA1	1794	MN611697.1	Erysiphe necator associated abispo virus 9 isolate PMS8_32 segment RNA1, complete sequence	99.00	0	95.76	1849	2887	2887
LS7	En_abispoV-RNA1	1710	MN611695.1	Erysiphe necator associated abispo virus 8 isolate PMS7_214 segment RNA1, complete sequence	97.00	0	95.90	1758	2689	2689
LS8	En_abispoV-RNA1	1029	MN611695.1	Erysiphe necator associated abispo virus 8 isolate PMS7_214 segment RNA1, complete sequence	100.00	0	95.14	1758	1624	1624
BS2	En_abispoV-RNA2	1850	MN611694.1	Erysiphe necator associated abispo virus 7 isolate PMS5_242 segment RNA2, complete sequence	99.00	0	94.49	1836	2824	2824
BS3	En_abispoV-RNA2	1572	MN611698.1	Erysiphe necator associated abispo virus 9 isolate PMS8_99 segment RNA2, complete sequence	100.00	0	94.02	1832	2383	2383
BS4	En_abispoV-RNA2	1828	MN611698.1	Erysiphe necator associated abispo virus 9 isolate PMS8_99 segment RNA2, complete sequence	100.00	0	94.31	1832	2802	2802
BS5	En_abispoV-RNA2	1825	MN611696.1	Erysiphe necator associated abispo virus 8 isolate PMS7_153 segment RNA2, complete sequence	99.00	0	94.89	1923	2850	2850
BS6	En_abispoV-RNA2	1744	MN611694.1	Erysiphe necator associated abispo virus 7 isolate PMS5_242 segment RNA2, complete sequence	100.00	0	94.09	1836	2651	2651
BS7	En_abispoV-RNA2	1831	MN611698.1	Erysiphe necator associated abispo virus 9 isolate PMS8_99 segment RNA2, complete sequence	99.00	0	94.54	1832	2826	2826
BS8	En_abispoV-RNA2	1836	MN611698.1	Erysiphe necator associated abispo virus 9 isolate PMS8_99 segment RNA2, complete sequence	99.00	0	94.54	1832	2826	2826
LS1	En_abispoV-RNA2	847	MN611698.1	Erysiphe necator associated abispo virus 9 isolate PMS8_99 segment RNA2, complete sequence	100.00	0	91.15	1832	1186	1186
LS2	En_abispoV-RNA2	1816	MN611694.1	Erysiphe necator associated abispo virus 7 isolate PMS5_242 segment RNA2, complete sequence	99.00	0	94.59	1836	2806	2806
LS3	En_abispoV-RNA2	1417	MN611696.1	Erysiphe necator associated abispo virus 8 isolate PMS7_153 segment RNA2, complete sequence	100.00	0	93.65	1923	2119	2119
LS4	En_abispoV-RNA2	145	MN611696.1	Erysiphe necator associated abispo virus 8 isolate PMS7_153 segment RNA2, complete sequence	100.00	5.00E-59	96.55	1923	241	241
LS6	En_abispoV-RNA2	1805	MN611698.1	Erysiphe necator associated abispo virus 9 isolate PMS8_99 segment RNA2, complete sequence	99.00	0	94.56	1832	2785	2785
LS7	En_abispoV-RNA2	563	MN611696.1	Erysiphe necator associated abispo virus 8 isolate PMS7_153 segment RNA2, complete sequence	100.00	0	94.49	1923	869	869
LS8	En_abispoV-RNA2	403	MN611696.1	Erysiphe necator associated abispo virus 8 isolate PMS7_153 segment RNA2, complete sequence	100.00	6.00E-172	94.29	1923	617	617
BS-9	BCTV	1110	KX867041.1	Beet curly top virus isolate CTS13-028, complete genome	100.00	0	96.31	3038	1842	1842
BS-3	BvANV-1	2272	MT227163.1	Beta vulgaris Alphanecrovirus-1 strain USA2020, complete sequence	100.00	0	99.91	3665	4185	4185

*BNYVV; Beet necrotic yellow vein virus, BSBMV; Beet soil-borne mosaic virus, BSBV; Beet soil-borne virus, BvSatV; Beta vulgaris satellite virus, En_abispoV; Erysiphe necator associated abispo virus, BCTV; Beet curly top virus, BvANV; Beta vulgaris alphanecrovirus
